# Identification of Putative Nuclear Receptors and Steroidogenic Enzymes in Murray-Darling Rainbowfish (*Melanotaenia fluviatilis*) Using RNA-Seq and *De Novo* Transcriptome Assembly

**DOI:** 10.1371/journal.pone.0142636

**Published:** 2015-11-23

**Authors:** Peter A. Bain, Alexie Papanicolaou, Anupama Kumar

**Affiliations:** 1 Commonwealth Scientific and Industrial Research Organisation (CSIRO), Division of Land and Water, Urrbrae, South Australia, Australia; 2 Commonwealth Scientific and Industrial Research Organisation (CSIRO), Division of Land and Water, Black Mountain, Australian Capital Territory, Australia; Glasgow Caledonian University, UNITED KINGDOM

## Abstract

Murray-Darling rainbowfish (*Melanotaenia fluviatilis* [Castelnau, 1878]; Atheriniformes: Melanotaeniidae) is a small-bodied teleost currently under development in Australasia as a test species for aquatic toxicological studies. To date, efforts towards the development of molecular biomarkers of contaminant exposure have been hindered by the lack of available sequence data. To address this, we sequenced messenger RNA from brain, liver and gonads of mature male and female fish and generated a high-quality draft transcriptome using a *de novo* assembly approach. 149,742 clusters of putative transcripts were obtained, encompassing 43,841 non-redundant protein-coding regions. Deduced amino acid sequences were annotated by functional inference based on similarity with sequences from manually curated protein sequence databases. The draft assembly contained protein-coding regions homologous to 95.7% of the complete cohort of predicted proteins from the taxonomically related species, *Oryzias latipes* (Japanese medaka). The mean length of rainbowfish protein-coding sequences relative to their medaka homologues was 92.1%, indicating that despite the limited number of tissues sampled a large proportion of the total expected number of protein-coding genes was captured in the study. Because of our interest in the effects of environmental contaminants on endocrine pathways, we manually curated subsets of coding regions for putative nuclear receptors and steroidogenic enzymes in the rainbowfish transcriptome, revealing 61 candidate nuclear receptors encompassing all known subfamilies, and 41 putative steroidogenic enzymes representing all major steroidogenic enzymes occurring in teleosts. The transcriptome presented here will be a valuable resource for researchers interested in biomarker development, protein structure and function, and contaminant-response genomics in Murray-Darling rainbowfish.

## Introduction

The Murray-Darling rainbowfish (*Melanotaenia fluviatilis* [Castelnau, 1878]; Atheriniformes: Melanotaeniidae) is a small-bodied freshwater fish endemic to the Murray-Darling basin, a region that produces approximately half of the Australia’s total irrigated agricultural output [1]. Due to its small body size, *M*. *fluviatilis* is readily maintained in laboratory aquaria and has been used to study the aquatic toxicology of organic and inorganic contaminants including pesticides and herbicides [2,3,4,5,6,7], heavy metals [8], and crude oil [9,10], and has been deployed in a field-based mobile laboratory to study effluent toxicity on-site [11]. Prominent secondary sexual characteristics combined with a relatively short maturation time make *M*. *fluviatilis* well-suited to investigating the sex-specific effects of endocrine disrupting chemicals (EDCs) on reproduction and sex-specific biomarkers [12,13,14,15,16,17,18,19].

The past two decades has seen increasing interest in the potential adverse effects of EDCs in aquatic organisms. The major focus to date has been on estrogenic EDCs, which have been shown to induce the expression of female-specific biomarkers in male fish and ultimately result in intersex condition in some species [20,21,22,23,24]. Small-bodied fish species have gained popularity as model organisms for EDC research in Asia (zebrafish and Japanese medaka), Europe (roach), and North America (fathead minnow). Through the use of estrogen-responsive biomarkers, most notably hepatic vitellogenin expression, variable inter-species sensitivity to natural and synthetic estrogens has been observed in laboratory-based tests [25]. In the case of xenoestrogens, inter-species differences sensitivity can be predicted using *in vitro* reporter assays [25] and are least partly explained by sequence diversity in the ligand binding domain of estrogen receptor α (ERα) [26], suggesting that characterising the phylogenetics and *in vitro* sensitivity of key receptors can provide insight into the potential susceptibility of a given fish species to the effects of EDCs. Since the environmental persistence of endocrine-active contaminants such as pharmaceuticals varies with temperature, sunlight and water chemistry [27] estimation of potential risks is likely to be improved through the use of endemic species as test organisms and by taking local environmental conditions into consideration during experimental design. While some progress has been made with regard to the effects of EDCs on endemic species in the southern hemisphere, a recent report highlighted the need for further research in native freshwater fish species in Australia [28].

A teleost-specific genome duplication (TSGD) event [29,30,31,32] is thought to have contributed to the extreme physiological and genetic diversity present in extant members of this lineage, which is estimated to comprise more than 26,000 species [33]. The maintenance and subsequent sequence divergence of gene pairs arising from the TSGD has in many cases resulted in the functional differentiation of paralogous genes. For example, two copies of the *ESR2* gene, encoding ERβ, have been maintained in most teleosts, with the two receptor subtypes ERβ1 and ERβ2 responding with different sensitivity to estrogens and xenoestrogens [34,35]. Many acanthomorpha (spiny-finned fishes including medaka and rainbowfish) are also known to express two androgen receptor (AR) subtypes, designated ARα and ARβ, while in the otomorpha group (which includes cyprinids such as zebrafish and roach) a single AR subtype similar to ARβ is present [36]. We have recently shown that ARα and ARβ from Murray-Darling rainbowfish exhibit differential sensitivity to both agonists and antagonists [37], suggesting that the divergence of AR paralogues, like in the case of ERβ, is sufficient to confer variability in ligand selectivity. Receptor sequences in other lineages have diversified further due to additional rounds of genome duplication–rainbow trout (*Oncorhynchus mykiss*) expresses two paralogous forms of ARβ [38] as a result of a postulated genome duplication specific to salmonids [39]. Differences in the number of paralogues of genes encoding steroid receptors, as well as their sequence diversity, contributes to inter-species differences in sensitivity. Indeed, in the case of ER-mediated biomarkers such as vitellogenin, sensitivity in response to exposure to 17α-ethinylestradiol has been shown to vary significantly in different species [25]. This concept can likely be extended to other nuclear receptors (NRs) and indeed to any receptor capable interacting with xenobiotics. For non-model organisms, the lack of readily available sequence data for such receptors hinders development of quantitative biomarker assays and functional studies of relevant proteins such as steroid hormone receptors. Due to the continuing cost reductions associated with next-generation sequencing, the most efficient means of generating such sequence data for non-model organisms is high-throughput mRNA sequencing (RNA-Seq), which provides data necessary for the rapid design and implementation of focused gene expression assays (qPCR) and custom microarrays [40,41] and enables protein functional studies to proceed quickly by providing coding sequence data required for cloning and in vitro expression [42].

In the present study, we sequenced messenger RNA (mRNA) from brain, liver and gonads of sexually mature male and female *M*. *fluviatilis* and prepared an annotated database of candidate protein-coding transcripts using a *de novo* assembly approach. The resulting transcriptome was found to be highly comprehensive, with full-length homologues present for the majority of genes predicted from the genome sequence of a taxonomically related teleost. Since one of our group’s major research interests is environmental endocrine disruption, we analysed the rainbowfish transcriptome to identify NRs and steroid biosynthetic enzymes that could potentially be involved in responses to EDCs. The results are presented here alongside an overview of the transcriptome as a whole.

## Results and Discussion

### Sequencing, transcript assembly and annotation

Poly-A^+^ messenger RNA from brain, liver and gonad tissue from one adult male and one adult female Murray-Darling rainbowfish was sequenced using the Illumina HiSeq 2000 platform. The use of a single individual of each sex was intended to reduce the likelihood of assembly errors due to intra-specific polymorphism. A total of 258,906,260 read pairs from all libraries (S1 Table) were subjected to quality control procedures and assembled using Trinity [43,44], yielding a total of 262,050 putative transcripts in 149,742 clusters (Table 1).

**Table 1:** Statistics of the transcriptome assembly.

**Trinity transcripts**	262,050
**Clusters**	149,742
**GC content (%)**	44.51
**Contig N50**	3,367
**Median contig length**	655
**Mean contig length**	1539
**Mean read length after QC**	85
**Mean fragment size**	137
**Total assembled bases**	403,186,007

148,394 likely open reading frames (ORFs) were identified using a Markov model-based approach to predict candidate protein-coding regions with a minimum length of 300 nucleotides. Redundancy in the complete set of ORFs was reduced by clustering sequences based on similarity of the deduced protein sequences, resulting in 43,841 non-redundant ORFs corresponding to a deduced amino acid sequence identity of ≥ 95%. Of these, 25,339 were deemed complete in terms of the coding sequence (i.e. beginning with a start codon and ending with a stop codon), with the remaining sequences partial at either the 5’ or 3’ ends. Deduced proteins were annotated using functional vocabularies associated with homologous sequences (HHblits e-value ≤ 1e-10) in the Swiss-Prot database [45], with assignment of putative functions derived from numerous sources including Gene Ontology [46], KEGG [47], and EggNOG [48]. Expression levels for each non-redundant ORF were estimated by estimating abundance according to read coverage as described in the methods section. The complete set of assembled transcripts along with JAMp-derived functional annotations and normalised expression data for the non-redundant protein-coding transcripts are available through a web interface (http://annotation.insectacentral.org/waite/). Assembled transcripts and raw reads are available from NCBI BioProject (http://www.ncbi.nlm.nih.gov/bioproject/), accession PRJNA282059. Lists of assembled transcripts, predicted coding regions and non-redundant deduced protein sequences are also available from the CSIRO Data Access Portal (http://dx.doi.org/10.4225/08/563BDB4789194).

### Assessment of the completeness of the transcriptome assembly

For the present study, we did not expect to identify a complete set transcripts spanning the entire genome due to the limited number of tissues sampled and the fact that we only sampled adult specimens maintained under laboratory conditions. However, we were interested in estimating the completeness of the assembly in terms of both the number of non-redundant protein-coding sequences present in the rainbowfish transcriptome that could be considered as homologous to sequences from known complete eukaryotic genomes, and the degree of coverage present for each homologue. We approached these questions using two methods, the first utilising a limited subset of highly conserved eukaryotic proteins [49] and the second involving the complete predicted proteome of a close taxonomic relative.

**Fig 1:**
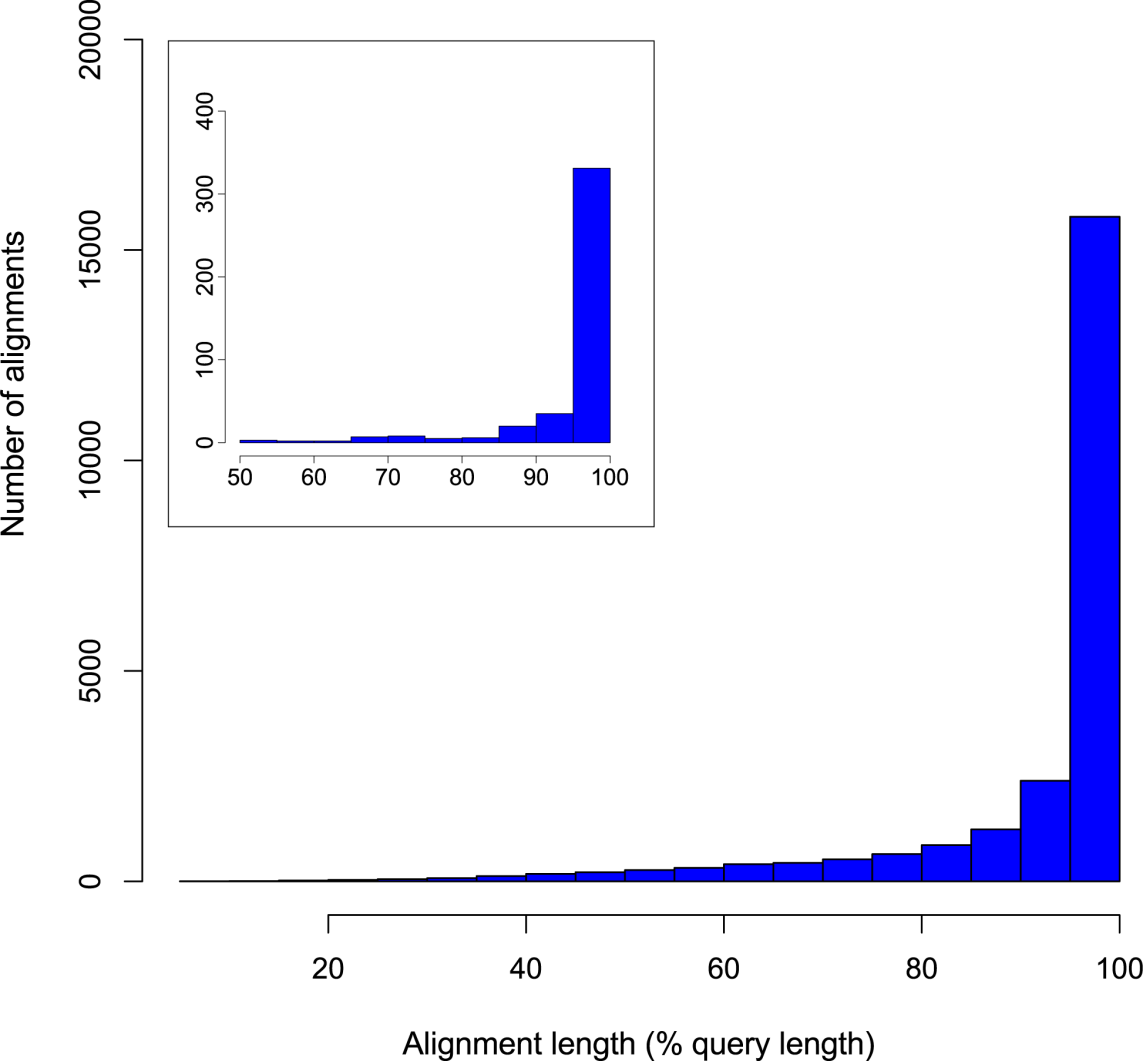
Analysis of the completeness of the rainbowfish transcriptome. Sequence similarity searches were conducted using the full set of predicted proteins derived from the Japanese medaka genome (*Oryzias latipes*; Ensembl release 76) as BLASTP queries against translated open reading frames from the Murray-Darling rainbowfish transcriptome. From 24,674 medaka query sequences, 23,601 high-quality (BLASTP e-value ≤ 1e-10) matches were obtained (95.7%) with a mean query coverage of 92.1%. Alignment length distributions are shown (bin size 5%), expressed as a proportion of medaka protein sequence lengths (% query coverage). **INSET:** A similar analysis conducted using the highly conserved subset of core eukaryotic genes (CEGs) from medaka.

Firstly, we identified rainbowfish sequences corresponding to a subset of ‘core eukaryotic genes’ (CEGs) using the CEGMA software package [49], which is commonly used to assess the completeness of draft genome sequence assemblies but has also been applied to transcriptome assemblies [50,51]. According to CEGMA analysis, 232 of 248 CEGs (93.55%) were present as complete sequences in the rainbowfish transcriptome, with at least partial matches present for 236 CEGs (95.16%). Because Japanese medaka (*Oryzias latipes*) is the closest relative of rainbowfish for whole-genome sequence data is available, we also used the medaka subset of CEG mappings (available from http://korflab.ucdavis.edu/Datasets/genome_completeness/) as queries to search for homologous sequences in the non-redundant set of predicted rainbowfish proteins using BLASTP [52]. Of the 421 medaka CEGs, 405 unique rainbowfish sequences exhibiting high similarity (expect value ≤ 1e-10) and minimum query coverage of 70% (referring to the length of the alignment as a proportion of the length of the query sequence) were obtained (96.2%; S2 Table). The mean query coverage of these matches was 95.83% and the mean sequence identity of the alignments was 90.4%. The query coverage distribution shown in Fig 1 (inset) suggested that the number of sequences exhibiting low query coverage, indicative of either partial ORFs or non-orthologous hits to highly conserved domains, was low. These data indicate that full-length homologues were present in the rainbowfish transcriptome assembly for the majority of CEGs mapped in the medaka genome.

Secondly, we identified rainbowfish protein sequences homologous (BLASTP e-value ≤ 1e-10) to the entire set of medaka proteins predicted from the *O*. *latipes* genome sequence (ftp://ftp.ensembl.org/pub/release-76/fasta/oryzias_latipes/pep/; Ensembl release 76). This revealed 23,601 rainbowfish sequences homologous to 24,674 medaka proteins (95.7%; S3 Table), with a mean query coverage 92.1%. Query coverage distributions are shown in Fig 1. These values indicate that despite the limited number of tissues sampled in the present study, the assembled transcriptome contains full-length orthologues for a large proportion of the proteins predicted from medaka genome.

### Identification of putative nuclear receptors in the rainbowfish transcriptome

The focus of the present study was to generate sequence data for key proteins of the reproductive endocrine system—primarily steroidogenic enzymes, nuclear receptors and the target genes thereof—to aid in the development of molecular methods for predicting of the potential impacts on fish health of various endocrine-active contaminants. Since the best-studied EDCs are modulators of estrogen and androgen receptors, obtaining full-length sequence data for all subtypes of these receptors in rainbowfish was a high priority, but we were also interested in NRs that could potentially be activated or inhibited by a diverse range of environmental contaminants of emerging concern, including active pharmaceutical ingredients.

The major source of pharmaceuticals in surface waters is sewage, which contains a wide range of pharmaceutical drugs and metabolites that are incompletely removed during treatment [53,54,55,56,57,58,59,60,61]. Many drugs designed to target NRs in humans have the potential to interact with fish NRs due to the relatively high degree of sequence conservation amongst vertebrates. Indeed, some pharmaceuticals have been shown to affect developmental and reproductive endpoints in fish. Examples include dexamethasone, a steroidal glucocorticoid receptor (GR) agonist that inhibits growth and development in fathead minnow [62] and 19-nortestosterone-derived synthetic progestogens, which exhibit androgenic activity *in vivo* [63,64,65] and are potent agonists of ARs from fathead minnow [66] and Murray-Darling rainbowfish [67]. Peroxisome proliferator-activated receptors (PPARs) are also drug targets, with PPARα and β ligands including lipid-lowering fibrates, and anti-diabetes drugs that target PPARγ. While little data is currently available regarding the effects of pharmaceuticals on fish PPARs, fibrates have been shown to activate PPARα and β from rainbow trout [68], and the anti-diabetic drug rosiglitazone is a weak agonist of PPARγ from sea bream [69]. Because NRs control numerous fundamental developmental and physiological processes in vertebrates and are amenable to modulation by xenobiotics in the aquatic environment, the functional characterisation of NRs in fish species used for toxicological research will significantly advance our understanding of the potential risks to fish health posed by various endocrine-active environmental contaminants.

**Fig 2:**
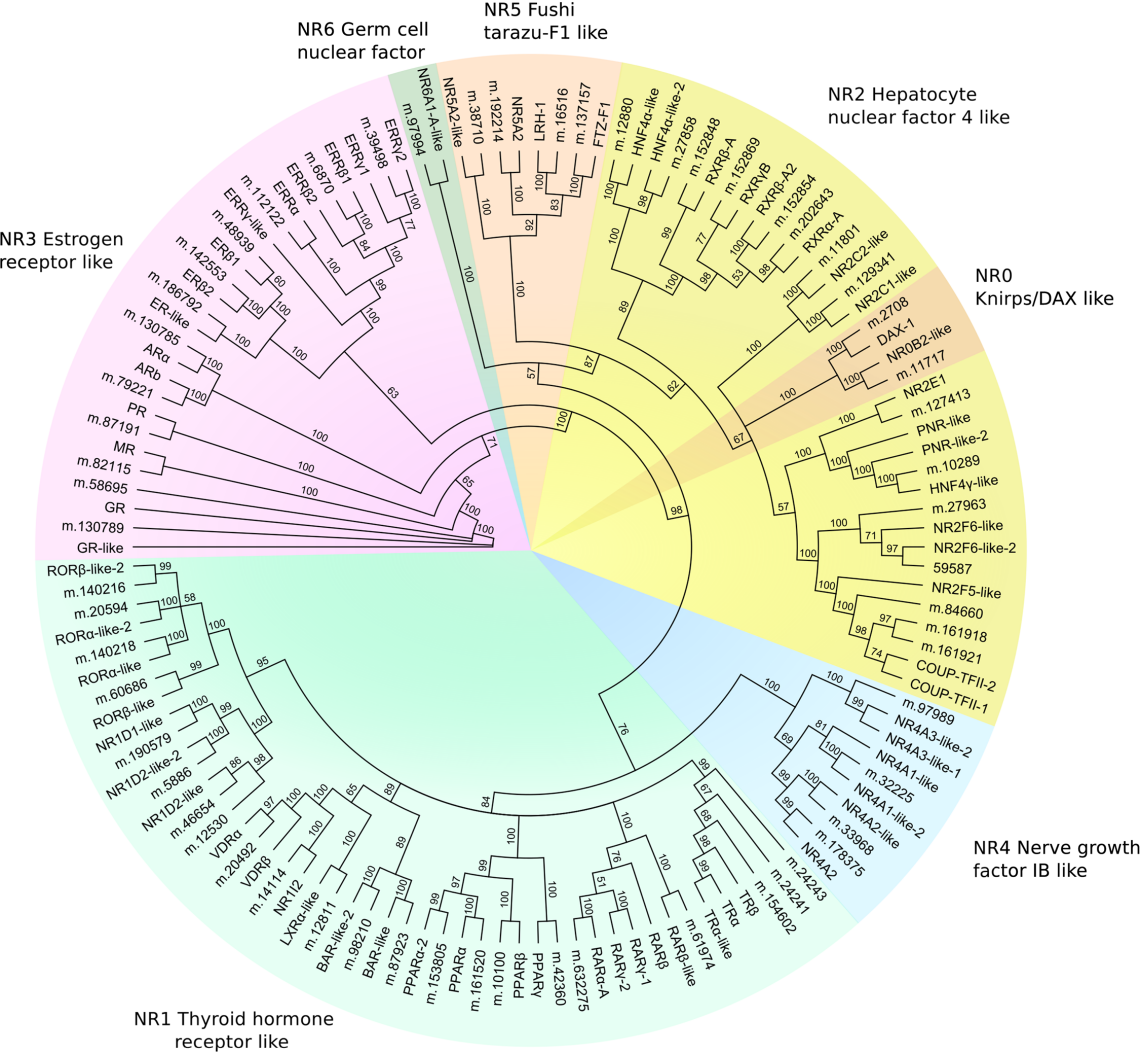
Phylogenetic analysis of candidate nuclear receptors from rainbowfish and nuclear receptors sequences from the Japanese medaka (*Oryzias latipes*) genome. Node labels indicate percent bootstrap support from 100 replications. Branch lengths are not to scale. Candidate nuclear receptors from the rainbowfish transcriptome are indicated by their coding sequence identifiers (m.*). Abbreviations: AR, androgen receptor; BAR, bile acid receptor; COUP-TF, chicken ovalbumin upstream promoter transcription factor; DAX-1, dosage-sensitive sex reversal, adrenal hypoplasia critical region, on chromosome X, gene 1; PNR, photoreceptor cell-specific nuclear receptor; ER, estrogen receptor; ERR, estrogen receptor-related; FTZ-F1, fushi tarazu factor 1; GR, glucocorticoid receptor; HNF, hepatocyte nuclear factor; LRH, liver receptor homologue; LXR, liver X receptor; MR, mineralocorticoid receptor; NR, nuclear receptor; PPAR, peroxisome proliferator-activated receptor; PR, progestin receptor; RAR, retinoic acid receptor; ROR, RAR-related orphan receptor; RXR, retinoid X receptor; TR, thyroid hormone receptor; VDR, vitamin D receptor. NRs not assigned a common name are denoted by their subfamily, group and member number, i.e. ‘NR5A2’ represents nuclear receptor subfamily 5, group A, member 2.

Canonical NRs are comprised of a number of functional domains necessary for their activity as ligand-activated transcription factors, including an amino-terminal activation domain, a DNA-binding domain (DBD), and a ligand-binding domain (LBD) (reviewed by Robinson-Rechavi et al., [70]). The most highly conserved regions are the DBD and the LBD, which hence can be used to annotate candidate NRs according to sequence similarity with known receptors. To identify putative NRs in the rainbowfish transcriptome, similarity searches were undertaken using the NCBI Batch CD-Search tool (http://www.ncbi.nlm.nih.gov/Structure/bwrpsb/bwrpsb.cgi), which searches the NCBI Conserved Domain database to identify putative functional domains in query sequences. Rainbowfish sequences exhibiting high-scoring matches to the both the DBD superfamily (Pfam cl02596; ‘NR_DBD_like’) and the LBD superfamily (Pfam cl11397; ‘NR_LBD’) were identified, resulting in a list of 66 candidate nuclear receptors (S4 Table). This list was manually curated and annotated using NRs predicted from the Japanese medaka genome (listed in KEGG BRITE ola03310) resulting in a set of 61 candidate rainbowfish NRs (S5 Table). To categorise the NRs into subfamilies [71], a phylogenetic analysis was undertaken based on a multiple alignment of DBD and LBD sequences from the putative rainbowfish and medaka NRs (Fig 2). This revealed that representatives for all seven NR groups were present in the rainbowfish transcriptome, with the NR1, NR2 and NR3 subfamilies comprising the majority of sequences. The mean query coverage of rainbowfish candidate NRs with respect to medaka NRs was 87.3% and the mean amino acid identity was 85.9%.

The availability of sequence data for rainbowfish nuclear hormone receptors will allow us and other researchers to establish *in vitro* concentration-response relationships for various endocrine-active compounds using reporter gene transactivation assays. *In vitro* reporter gene assays have been demonstrated to correlate well with *in vivo* sensitivity to endocrine disruptors such as xenoestrogens [25,35]. Based on data generated as part of the present study, we have already successfully amplified full-length coding sequences for rainbowfish ARα and ARβ and confirmed their function as androgen-dependent transcription factors capable of transactivation through canonical androgen response elements [37]. Our *in vitro* study showed that rainbowfish ARα and ARβ exhibit markedly different sensitivities to both synthetic androgens and anti-androgens, indicating that the divergence of paralogous duplicates arising from the TSGD is sufficient to confer differential responses to selected ligands. We have also found that synthetic progestins derived from 19-nortestosterone are potent agonists of rainbowfish ARα and ARα but do not activate the rainbowfish progestin receptor [67], adding to recent data indicating that fathead minnow AR is activated strongly by progestins [66] and providing further mechanistic evidence that androgen receptor activation underlies the effects observed in various species exposed to progestins *in vivo* [63,64,65,72,73].

### Identification of candidate steroidogenic enzymes

Normal functioning of endocrine systems in fish can be altered by EDCs not only by direct interaction with hormone receptors, but also via the binding and inhibition of steroidogenic enzymes [74], most of which belong to the cytochrome P (CYP)450 superfamily of haem-containing mono-oxygenases. One of the best studied examples in the context of EDCs is the inhibition of the enzyme responsible for conversion of androgens to estrogens, CYP19 aromatase, by azole compounds including fungicides [75]. Mechanistic studies using the specific aromatase inhibitor, fadrazole, have shed light on the dramatic effects aromatase inhibitors can elicit in fish [76]. Exposure to fadrazole during a developmental window critical for sexual differentiation can result in the complete masculinisation of genetically female chinook salmon [77]. Teleosts may be particularly susceptible to aromatase inhibitors due to presence of an additional genetic locus encoding a brain-specific subtype denoted CYP19A1b, expressed at high levels during early development [78].

Changes in the expression of steroid biosynthetic genes can also occur in response to EDCs and is considered one of the causative mechanisms underlying altered plasma steroid levels in exposed organisms [74,79,80,81,82]. Specific examples include the induction of aromatase gene expression in response to estrogens [79,83] and increased expression of 20β-hydroxysteroid dehydrogenase (20β-HSD), a key enzyme in the synthesis of the progestins in fish, in response to anti-androgens [84].

**Fig 3:**
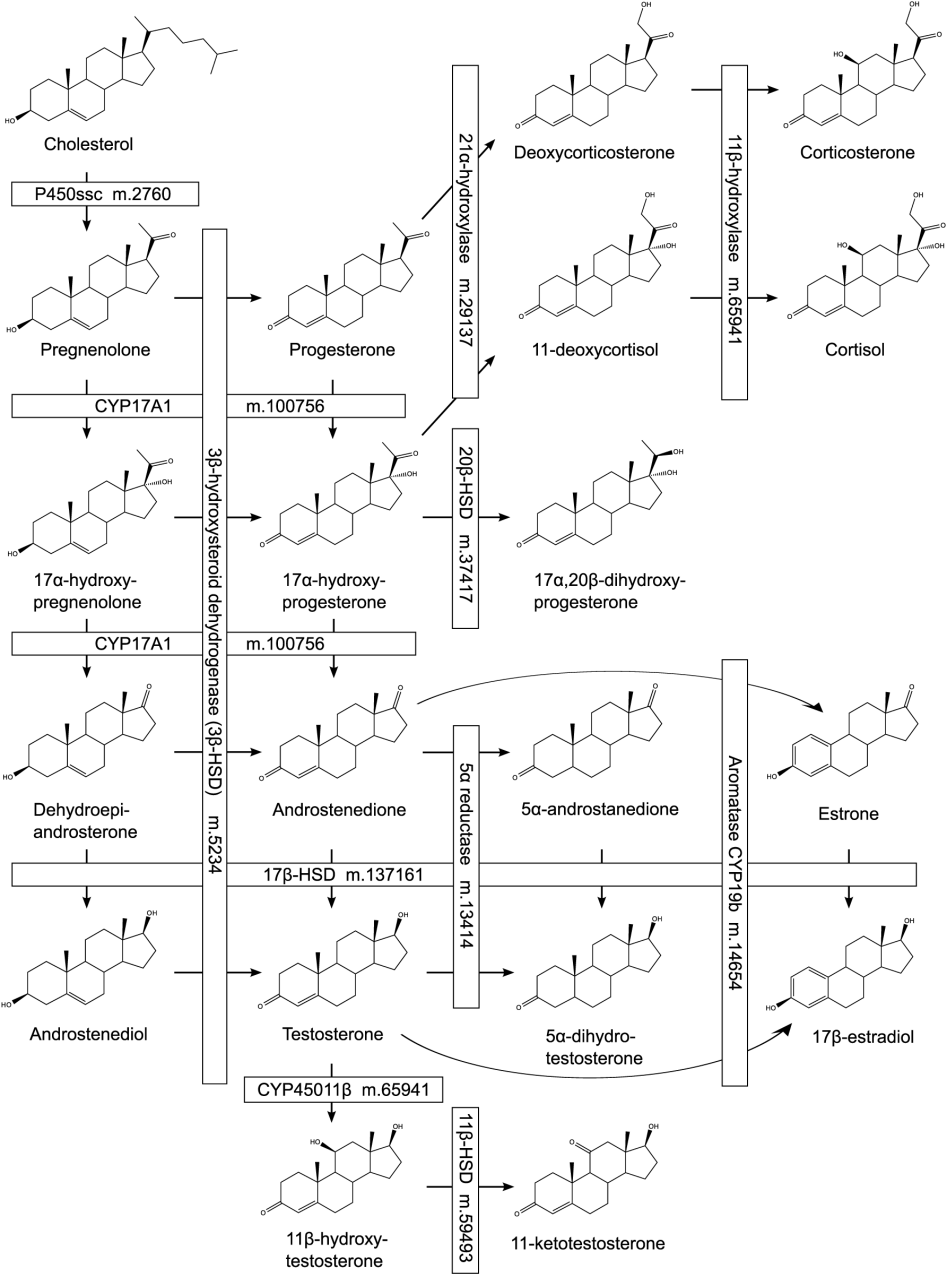
Schematic diagram of likely steroidogenic pathways in Murray-Darling rainbowfish. ORF identifiers corresponding to deduced proteins displaying high similarity to biosynthetic enzymes are shown alongside enzyme descriptions at each step of the biosynthetic pathway. Adapted from Häggström and Richfield [85] with reference to known teleost steroidogenic pathways [86,87,88].

To identify candidate steroid biosynthetic enzymes in the rainbowfish transcriptome, *O*. *latipes* proteins annotated as members of steroidogenic pathways (KEGG pathway ola00140 ‘steroid hormone biosynthesis’) were used as query sequences for similarity searches of the complete set of translated rainbowfish ORFs. This resulted in a list of 41 unique high-quality matches (maximum e-value of 2.8e-32) with a mean query coverage of 92.46% (S6 Table). At least one full-length match was present for each of the major enzymes involved in steroidogenesis in teleosts. To demonstrate that the majority of key enzymes involved in steroid hormone biosynthesis were identified in the present study, rainbowfish ORF identifiers corresponding to the best match for each enzyme are listed alongside enzyme descriptions in a schematic diagram of steroid biosynthesis in teleosts (Fig 3; adapted from Häggström and Richfield [85] with reference to pathways described in [86,87,88]).

The presence of a 5α reductase in Murray-Darling rainbowfish transcriptome raises the question of whether the androgen 5α-dihydrotestosterone (DHT) may be present at physiologically relevant levels in this species, as has been reported for fathead minnow (*Pimephales promelas*) [87,89]. We have observed using in vitro reporter assays that DHT is comparable in potency with 11-KT as an agonist of both ARα and ARβ [37], suggesting that endogenous DHT, if present, may be an important androgen in *M*. *fluviatilis*.

### Identification of transcripts for estrogen-responsive genes

A number of genes known to be positively regulated at least in part by ERs have been validated as biomarkers of (xeno)estrogen exposure in male or juvenile fish [90,91]. Vitellogenin (VTG) and choriogenins (ChgH and ChgL), precursors to yolk and egg envelope proteins, respectively, are the most widely employed biomarkers of ER activation [91,92]. Plasma VTG levels are often determined using specific antibodies, but since the site of VTG synthesis is the liver, hepatic mRNA levels for VTG are closely correlated with plasma protein and can be utilised as a reliable surrogate if antibodies for a particular species are not available. Expression of the *esr1* gene in fish (encoding ERα) is also induced in response to estrogens [93,94,95], indicating an auto-regulatory mechanism. ERα mRNA is also used to infer exposure to (xeno)estrogens in male fish, since increased levels have been positively correlated with histological changes consistent with intersex condition [24]. We have previously developed quantitative reverse-transcription PCR (qRT-PCR) assays for selected ER-regulated transcripts in rainbowfish, including VTG [16], choriogenin L (ChgL [18]), and ERα [16], but to date only ERα has been fully sequenced for this species.

To identify putative estrogen-responsive genes in the rainbowfish transcriptome, we used a subset of estrogen-responsive genes from medaka as BLAST queries. Full-length sequences were found for three VTG subtypes (denoted VTG A, B and C), ERα, ERβ1, ERβ2, choriogenin H (ChgH) and ChgL (Table 2). Multiple putative splicing variants of similar length were assembled for VTG A and B. ERβ1 and ERβ2 sequences required manual curation in order to obtain full-length putative coding sequences. In the case of ERβ1, an apparent frameshift was present in the predicted coding sequence. In an attempt to resolve a complete coding sequence, we generated an alternative assembly using only ovary-derived reads, which was found to contain an ERβ1-like ORF of similar length to published sequences from other teleosts. For ERβ2, a suspected assembly error was apparent in a T-rich region, resulting in a frameshift in the predicted coding sequence. This error was confirmed by sequencing a PCR product generated using primers spanning the CDS, and a complete coding sequence was thus btained. Full-length coding sequences for estrogen-responsive transcripts generated in the present study will facilitate further gene expression and functional studies.

**Table 2:** Rainbowfish transcripts with high similarity to estrogen-responsive genes from related teleosts.

Description	Rainbowfish transcript|ORF	Deduced protein length (aa)	Closest BLAST match (GenBank non-redundant protein sequences, *M*.*fluviatilis* omitted)	Length of closest match (aa)	GenBank Accession
Vitellogenin A	comp98304_c0_seq1|m.58126, comp98304_c0_seq2|m.58130, comp98304_c0_seq3|m.58134	1,717, 1,699, 1,690	vitellogenin [*Gambusia affinis*]	1,695	BAD93697
Vitellogenin B	comp102542_c3_seq1|m.120426, comp102542_c3_seq2|m.120431, comp102542_c3_seq3|m.120436, comp102542_c3_seq4|m.120441	1,733, 1,717, 1,713, 1,709	vitellogenin Ab [*Dicentrarchus labrax*]	1,692	AFA26670
Vitellogenin C	comp42500_c0_seq1|m.2380,	1,244	vitellogenin C [*Dicentrarchus labrax*]	1,275	AFA26671
Estrogen receptor α	comp105150_c0_seq9|m.186792	611	estrogen receptor alpha [*Odontesthes bonariensis*]	602	ABY19510
Estrogen receptor β1	transcript from alternative assembly (ovary)	559	estrogen receptor beta 1 [*Odontesthes bonariensis*]	558	ABY19511
Estrogen receptor β2	comp103476_c2_seq6|m.142553 (manually edited to remove likely assembly error in poly-U tract and verified by sequencing)	665	estrogen receptor beta 2 [*Oreochromis niloticus*]	666	NP_001266406
Choriogenin H	comp78177_c1_seq1|m.9381	466	choriogenin H minor [*Fundulus heteroclitus*]	452	BAJ07537
Choriogenin L	comp78177_c1_seq1|m.9382	440	L-SF precursor [*Oryzias latipes*]	420	NP_001098273

### Identification of ERα transcript variants

Alternative splicing of mRNA resulting in exon skipping or variable first or last exons generates protein isoforms with altered function compared to the canonical splice form (reviewed by Black et al. [96]). It has been estimated from high-throughput sequencing that approximately 95% of multi-exon genes in the human genome undergo alternative splicing [97], suggesting that the spectrum of protein products encoded by higher eukaryotic genomes greatly outnumbers the gene count. The occurrence of multiple ERα transcript variants derived from alternative splicing of pre-mRNA has been reported in a number of teleosts [98,99,100,101,102,103,104,105,106] and can contribute to environmental adaptations. For example, in killifish adapted to high levels of xenoestrogens in a contaminated waterway, alternative mRNA splicing has been proposed as one mechanism by which estrogenic responses are dampened [98]. RNA-Seq is a powerful tool for discovery of alternatively spliced transcripts [97].

According to comparative studies, Trinity is the leading *de novo* transcript assembly software for the reconstruction of mRNA splicing variants [107,108,109], prompting us to investigate whether rainbowfish ERα mRNA splicing variants were accurately predicted by Trinity in the present study. Sequences in the Trinity transcript cluster *comp105150_c0* exhibited high similarity to teleost ERα coding sequences and contained 12 transcripts with ORFs of varying lengths due to disruption frameshifts arising from apparent alternative splicing or the presence of unspliced introns. Possible donor and acceptor dinucleotides [110] could be identified in those variants with large apparent inserts (data not shown) suggesting the presence of introns, possibly attributable to the co-purification of immature RNA in the samples used for sequencing.

Three of the transcripts, *comp105150_c0_seq9*, *comp105150_c0_seq11* and *comp105150_c0_seq12*, encoded putative full-length ORFs typical of teleost ERα and were therefore chosen for further study. Interestingly, all putative transcripts in the cluster contained identical 5’ ends, suggesting that exon skipping may underlie the apparent transcript diversity in the 5’ region–*seq9* and *seq11* differed from *seq12* by the inclusion of additional sequence following the first exon, with a further insertion predicted for *seq9* relative to that predicted for *seq11*. The protein encoded by *seq9* and *seq11* differs from the *seq12*-encoded protein by only two amino acids at the amino terminus, (‘M**LL**RQS…’ in Seq9 and 11 and ‘M**FK**RQS…’ in Seq12). Interestingly, these sequences are consistent with two conflicting medaka *esr1* gene models, one of which (GenBank AB033491.1) predicts a structure similar to *comp105150_c0_seq12*, while the other (ENSORLG00000014514, Ensembl medaka release 78) predicts an exon structure and CDS similar to *comp105150_c0_seq9* and *seq11* (with the exception of putative first exon, which is omitted in the Ensemble gene model, with exon 1 annotated from the start of the CDS). Like the predicted rainbowfish proteins, translation of two medaka coding sequences yields two proteins differing only in first few amino acids—‘M**LL**RQS…’ in the longer form (UniProt H2MHS4, identical to the translated CDS from ENSORLG00000014514) and ‘M**SK**RQS…’ in the shorter form (UniProt P50241, identical to the translated CDS from GenBank AB033491.1). Interestingly, two similar ERα variants have been proposed in guppy (*Poecilia reticulata*), with deduced amino acid sequences differing by only two residues (‘M**LL**RQN…’, GenBank AB621910; and ‘M**YK**RQN…’, GenBank XP_008395912).

ERα splicing variants sharing a common first exon have also been described in killifish (*Fundulus heterclitus*) and have been postulated to result from the splicing of exon 1 to two alternative second exons denoted 2a or 2b, which differ in length by approximately 200 bases [98]. The longer killifish ERα transcript is similar to the *comp105150_c0_seq9-seq11* transcript described in the present study. The shorter killifish ERα transcript, first described by Urushitani et al. [111] and later confirmed by Greytak and Callard [112], contains an ORF of identical length to that in the longer transcript but encodes a protein that, similar to the putative rainbowfish isoforms and the medaka gene models, differs by only a few amino acids immediately following the initiator methionine (‘M**LLR**Q…’ and ‘M**YKG**Q…’ from the long and short transcripts, respectively). Both the long and the short killifish transcripts have two possible translational start codons–the second located 141 bases downstream from the first, which would result in the omission of 47 amino acids from the amino terminus. Translation from the second ATG of either transcript isoform would result in a receptor similar to the short form of rainbow trout ERα (ERα_S_), which displays higher ligand-independent transactivation activity than the long form (ERα_L_) [100], due to the lack of a possible ‘suppression’ domain.

**Fig 4:**
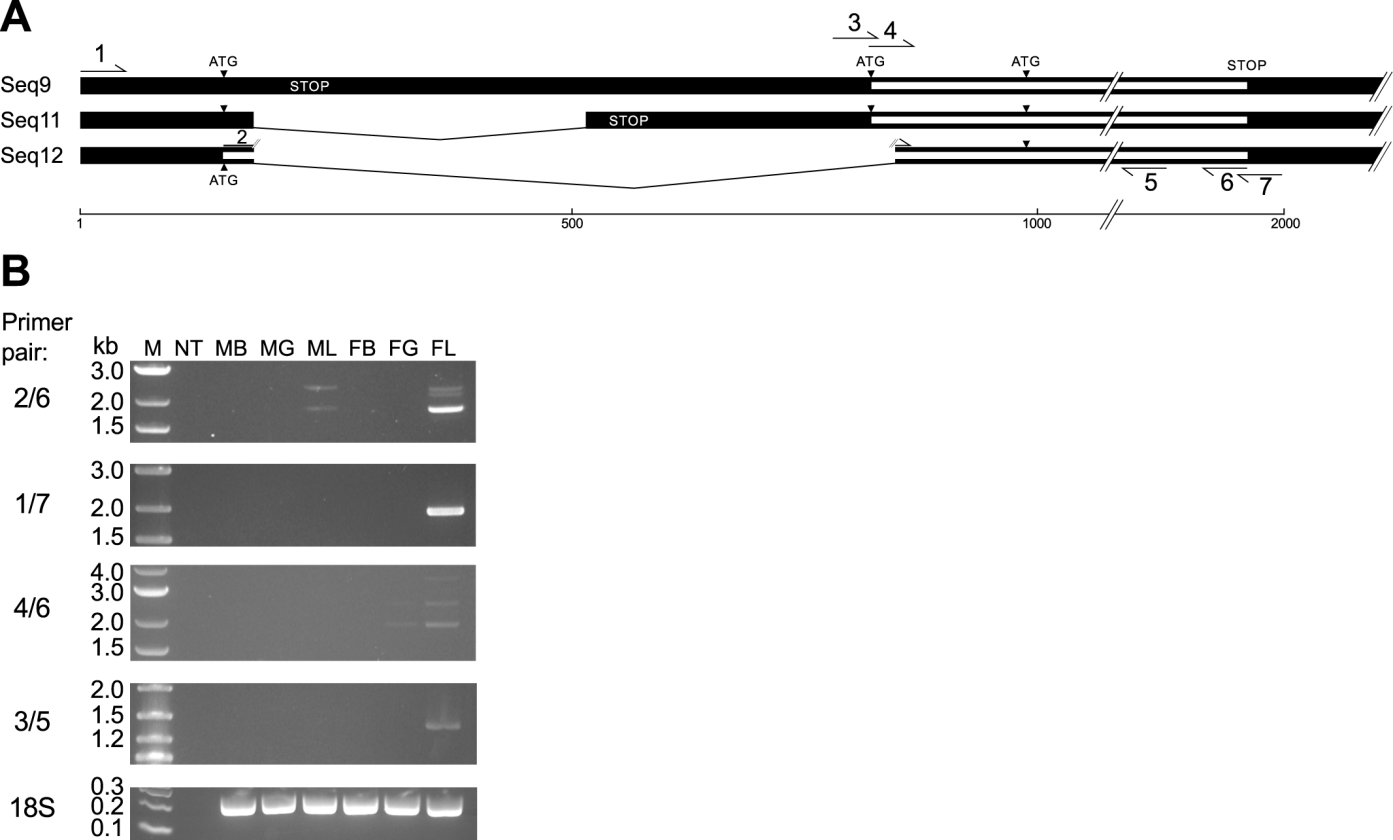
Confirmation of selected transcript variants of a putative rainbowfish ERα cluster by endpoint reverse-transcriptase PCR (RT-PCR). (A) Schematic diagram of three transcripts in the cluster–*comp105150_c0_seq9*, *comp105150_c0_seq11* and *comp105150_c0_seq12*, showing binding sites for the PCR primers listed in Table 4. (B) Agarose gel electrophoresis of RT-PCR products generated using the indicated primer pairs. Primers targeting 18S rRNA were used to confirm the quality of cDNA in all samples. Abbreviations: kb, kilobases; M, marker; NT, no-template control; MB, male brain; MG, male gonad; ML, male liver; FB, female brain; FG, female gonad; FL, female liver; 18S, 18S rRNA.

To confirm expression of the predicted rainbowfish ERα transcripts, we performed a series of end-point (non-quantitative) PCR reactions using primers targeting specific transcript variants (Fig 4A and Table 3). PCR products for the coding regions of both *seq9-seq11* and *seq12* were successfully amplified (Fig 4B) confirming that these coding variants were expressed to some degree. Both transcripts were expressed almost exclusively in female liver, with the *seq12* variant expressed at higher levels than *seq9-seq11* (although it should be noted that no biological replication was used in the present study and these results are from one male and one female individual fish). Additional bands appear at higher molecular weight may correspond to immature mRNA containing unspliced introns apparent in other sequences in the cluster (*seq1-8* and *seq10*). The solitary band resulting from primer pair 1/7 indicates that the *seq12* transcript is the predominant isoform. In fact, alignment of the rainbowfish transcript variants with the medaka *esr1* gene sequence (S1 Fig) shows that rainbowfish *seq9* may represent immature RNA, since the entire 5’ UTR aligns well with the medaka genome sequence, while the predicted *seq11* may constitute an shorter transcript derived from exon skipping as predicted for *esr1* in the draft guppy genome (Entrez Gene ID: 103457510). No short variants similar to the killifish ERα variants derived from exon skipping [98] were detected using RNA-Seq or RT-PCR in the present study.

**Table 3:** Primers used for RT-PCR analysis of rainbowfish ERα 5’-end variants.

**Primer number**	**Target transcript**	**Sequence (5’-3’)**
1	Seq9/11/12	CACCATGATTATTGATTCGGC
2	Seq12	ATGTTTAAGAGGCAGAGCCTG
3	Seq9/11	GCTGTGATGTTGCTCAGGCAG
4	Seq9/11	ATGTTGCTCAGGCAGAGCCT
5	Seq9/11/12	GCAGAAAGAAAAGGCACCAG
6	Seq9/11/12	TCATAGTGCGTGGGCGCA
7	Seq9/11/12	CCATCTCATAGTGCGTGGGC

### Transcripts encoding CYP450-family enzymes

In addition to their role in steroidogenesis, CYP450-family enzymes participate in the oxidative metabolism of a wide variety of endogenous and exogenous chemicals. Vertebrate CYP450s are classified into 18 families with varying substrate specificity [113]. Members of families CYP1, CYP2 and CYP3 are notable for their ability to metabolise xenobiotics such as polycyclic aromatic hydrocarbons (PAH)s, dioxins and polychlorinated biphenyls (PCBs). Elevated expression of CYP1A subfamily isoforms in fish liver tissue is a widely utilised biomarker for this class of environmental contaminants (reviewed by Bucheli and Fent [114]). The zebrafish (*Danio rerio*) genome contains 94 genes spanning the 18 vertebrate CYP450 families, including an apparent expansion of the CYP2 family to 47 members compared to 16 members in humans, with many of the zebrafish genes present on duplicated genomic regions arranged in tandem [115]. The genome of the pufferfish (*Takifugu rubripes*) encompasses 54 CYP450 genes from 17 families, including at least 24 putative CYP2-family sequences [116].

To identify full-length rainbowfish transcripts encoding putative CYP450-family enzymes, we retrieved zebrafish CYP450 protein sequences from the Ensembl zebrafish genome assembly Zv9 associated with PFAM family PF00067 (cytochrome P450) and performed a BLASTP similarity search against the complete list of predicted rainbowfish proteins. This resulted in 52 unique hits with at least one high-scoring match (maximum e-value for all hits was 3.06e-25) present for each of the 18 vertebrate CYP450 families (S7 Table). The mean query coverage was 90.76%. Like other teleosts, the CYP2 family had the largest number of representatives in the rainbowfish transcriptome, with 15 putative coding sequences. Given the high number of CYP2-family genes present in fugu [116] and zebrafish [115], it is possible that additional rainbowfish CYP2 transcripts were not detected in the current study due to low-level expression under normal laboratory conditions or in the tissues sampled. A candidate rainbowfish ORF encoding a protein with 70.6% amino acid identity to zebrafish CYP1A and query coverage of 98.85% was identified (*comp94347_c0_seq1|m*.*33721*), which may be suitable for use as a biomarker of compounds that induce the adaptive stress response.

### Transcripts involved in the xenobiotic response pathway

PCBs, PAHs and dioxins can induce potent xenobiotic defence responses in fish by binding to and activating the aryl hydrocarbon receptor (AhR). The functionality of AhR is unique in that it is the only ligand-activated member of the Per-ARNT-Sim family of basic-helix-loop-helix (bHLH) transcription factors [117,118]. Ligand-bound AhR is translocated to the nucleus, interacting with the Ah receptor nuclear translocator (ARNT) to induce the expression of target genes such as CYP1A by binding to dioxin response elements located in their proximal promoter regions [119]. The number and diversity of AhR genes in teleosts is considerable, with numerous subtypes of two distinct AhR genes denoted AhR1 and AhR2 expressed by some species [120,121,122]. For example, Fugu (*Takifugu rubripes*) expresses two AhR1 subtypes (*ahr1a* and *1b*), and three AhR2 subtypes (*ahr2a*, *2b*, and *2c*) [121,123]. In zebrafish, AhR1A is not capable of activating transcription through known response elements and thus may not be functional, while AhR1B and AhR2 are functional [123]. Zebrafish AhR2 binds TCDD and transactivates genes via AhR-responsive elements, while AhR1B is capable of transactivation but may be constitutively active since it does not appear to be TCDD-responsive [123].

To identify candidate members of the AhR pathway in the rainbowfish transcriptome, deduced protein sequences associated with the GO term ‘aryl hydrocarbon receptor activity’ (GO: 4874) were extracted from the JAMp database using the keyword search function in the web interface. Putative descriptions were inferred by performing individual BLASTP searches against the GenBank non-redundant protein database (S8 Table). The list of rainbowfish proteins included a partial ORF similar to teleost AhR1, a complete ORF encoding a protein with high similarity to teleost AhR2 sequences, and a number of full-length ORFs with high similarity to various isoforms of teleost ARNT (Table 4). These sequences could be useful for designing gene expression assays for biomarkers related to xenobiotic metabolism in Murray-Darling rainbowfish exposed to environmental contaminants such as dioxins, PCBs and PAHs, while the full-length coding sequence for an AhR2A homologue could be used to develop xenobiotic-responsive reporter gene assays capable of screening environmental samples for such contaminants.

**Table 4:** Transcripts involved in aryl hydrocarbon receptor (AhR)-mediated adaptive stress responses. Transcripts were classified into two functional groups–the nuclear AhR receptor complex (GO: 0034753) or AhR target genes comprising selected transcripts strongly upregulated by 2,3,7,8-Tetrachlorodibenzo-p-dioxin selected from Li et al. [124] and Watson et al. [125].

Putative description	Transcript/CDS identifier	Deduced protein length (aa)	Closest match (GenBank non-redundant protein sequences, *M*.*fluviatilis* omitted)	Length of closest match (aa)	Accession
AhR receptor complex					
Aryl hydrocarbon receptor 1A (AhR1A)	comp328301_c0_seq1|m.267373	268	aryl hydrocarbon receptor 1 [*Fundulus heteroclitus*]	944	AAR19369
Aryl hydrocarbon receptor 2A (AhR2A)	comp94961_c0_seq1|m.36116	914	aryl hydrocarbon receptor 2 [*Pagrus major*]	990	BAE02825
Aryl hydrocarbon receptor nuclear translocator (ARNT)	comp106403_c1_seq1|m.227926	638	PREDICTED: aryl hydrocarbon receptor nuclear translocator-like protein 1-like isoform X2 [*Oreochromis niloticus*]	638	XP_005449385
Aryl hydrocarbon receptor repressor (AhRR)	comp89767_c1_seq1|m.20660	639	PREDICTED: uncharacterized protein LOC100696058 [*Oreochromis niloticus*]	694	XP_003460145
AhR target genes					
Cytochrome P450 1A (CYP1A)	comp94347_c0_seq1|m.33721	421	Cytochrome P450 1A [*Poecilia vivipara*]	521	AFN02446
Cytochrome P450, family 1, subfamily B, polypeptide 1 (CYP1B1)	comp83107_c0_seq4|m.12292	583	PREDICTED: cytochrome P450 1B1-like [*Stegastes partitus*]	537	XP_008278388
Cytochrome P450, family 1, subfamily D, polypeptide 1	comp81594_c0_seq2|m.11394	537	PREDICTED: cytochrome P450 1A1-like [*Poecilia formosa*]	535	XP_007564479
Stromal cell-derived factor 2 (SDF2)	comp88906_c0_seq1|m.19082	245	PREDICTED: stromal cell-derived factor 2-like [*Neolamprologus brichardi*]	226	XP_006784901
Heat-shock protein A5 (HSPA5) / Glucose-regulated protein, 78 kDa (GRP78)	comp99062_c0_seq1|m.65001	564	Glucose-regulated protein 78 [*Paralichthys olivaceus*]	654	ABG56392
Aldehyde dehydrogenase 3 family, member A1 (ALDH3A1)	comp101664_c3_seq2|m.102761	532	PREDICTED: aldehyde dehydrogenase, dimeric NADP-preferring-like [Stegastes partitus]	509	XP_008297392
Nuclear factor erythroid 2-related factor 2 (Nrf2)	comp93427_c0_seq1|m.30054	617	PREDICTED: nuclear factor erythroid 2-related factor 2 [*Stegastes partitus*]	613	XP_008304424
NADPH:quinone oxidoreductase 1 (NQO1)	comp95672_c0_seq1|m.40009	272	NAD(P)H dehydrogenase quinone 1 [*Fundulus heteroclitus*]	277	ABD43173
Fibroblast growth factor 13 (FGF13)	comp105488_c2_seq1|m.196118	247	PREDICTED: fibroblast growth factor 13-like isoform X1 [*Oreochromis niloticus*]	246	XP_003445268

### Conclusions

Here we describe the sequencing, assembly, analysis and annotation of the Murray-Darling rainbowfish protein-coding transcriptome from adult male and female brain, liver and gonads. The rainbowfish transcriptome contains full-length orthologues for more than of 95% of the proteins predicted from the medaka genome, a taxonomically related species. The assembly therefore is likely to comprise not only a comprehensive repertoire of transcripts involved in steroid hormone biosynthesis and activity, but also the vast majority of transcripts expressed in adult liver, brain and gonads. This represents a valuable resource for researchers interested in protein structure and function, molecular toxicology, contaminant-response genomics and population genetics of the *Melanotaenia* genus, which is widely distributed throughout Australasia. It is becoming evident that genetic variability underlies the observed differences in inter-species responses to endocrine-active environmental contaminants. Thus, in aquatic environments impacted by such contaminants the responses observed in a single teleost species cannot be assumed to be representative of the potential impacts on fish in general and it may be more appropriate to characterise responses in numerous endemic species. Methods for rapidly obtaining sequence data for the nuclear receptors that largely define these interspecies differences will undoubtedly assist research efforts in this area. The methodology used in the present study is generally applicable and adds to the body of work demonstrating of the efficacy of RNA-Seq and *de novo* assembly in the rapid generation of protein-coding sequence data.

## Materials and Methods

### Animals

Sexually mature male and female *M*. *fluviatilis* were obtained from a commercial supplier (Aquarium Industries, NSW, Australia). Fish were maintained at 22°C under 16 h light / 8 h darkness photoperiod and fed dried fish flakes. The study was conducted with the approval of the Animal Ethics Committee of the Commonwealth Scientific and Industrial Research Organisation’s Division of Food and Nutritional Sciences (reference #774-12/13). One male (2.91 g, fork length 65 mm) and one female (5.1 g, fork length 80 mm) were anaesthetised using MS-222 prior to euthanasia by decapitation. Brain, liver and gonads were excised immediately and snap-frozen in liquid nitrogen.

### RNA isolation and sequencing

Total RNA was purified from approximately 15 mg liver or gonad tissue using the RNeasy® Mini Kit (QIAGEN Pty. Ltd., Australia). Total RNA from whole brain was purified using the RNeasy® Lipid Tissue Kit (QIAGEN Pty. Ltd., Australia). On-column DNase treatments were included during purification. Total RNA concentration and purity were estimated using a NanoDrop 1000 spectrophotometer (Thermo Scientific). RNA integrity was confirmed using the Bioanalzyer 2100 (Agilent).

Sequencing was undertaken by a service provider (Australian Genome Research Facility, Melbourne, Australia). Library preparation was performed using Illumina TruSeq reagents and sequencing was conducted using the HiSeq2000 platform. Briefly, RNA quality was determined using a Bioanalyzer 2100 (Agilent) before isolation of polyA^+^ mRNA, fragmentation, and construction of cDNA libraries using the Illumina TruSeq kit according to the standard protocol. 100-cycle paired-end sequencing was undertaken using two lanes of an Illumina HiSeq2000 instrument, with three libraries multiplexed per lane.

### Transcript assembly and annotation

For each library, average per-base quality and positional base composition were determined using FastQC (http://www.bioinformatics.babraham.ac.uk/projects/fastqc/). Bases with phred+33 quality scores of below 10 were trimmed from the 3’ end of each read using Trimmomatic [126]. Bases displaying obvious compositional bias (this comprised the initial 14 bases of each read according to summary graphs) were removed from all reads. Despite reports that compositional bias at the beginning of Illumina reads can be attributed to unexpected weighting of base composition in priming hexamers [127], we opted for a conservative approach since the overall sequencing quality and yield were high.

Trimmed reads were assembled into putative transcripts using Trinity [44] (http://trinityrnaseq.github.io/), release r2012-06-08. Default values were used for the assembly parameters [43], with the exception that the ‘—min_kmer_cov’ option defining the kmer coverage required for extension of contig ends was set to 2 in order to prevent contig extension by singleton kmers. 300 gigabytes of memory was allocated to the program, which was run using a high-memory server at the BRAEMBL high-performance computing facility, ‘Barrine’ (University of Queensland, St Lucia, Australia). To estimate fragment size of the paired-end libraries, reads were aligned against assembled transcripts using the default alignment options in RSEM (http://deweylab.biostat.wisc.edu/rsem/), with fragment size distributions visualised using the included R script.

Protein-coding regions were predicted using TransDecoder (http://transdecoder.github.io/) with the default parameters. TransDecoder utilises a Markov model trained on the longest open reading frames (ORFs) to predict a set of likely protein coding sequences. In cases where assembled transcripts contained more than one ORF, all ORFs were retained with the exception of those occurring completely within a larger ORF. To calculate read counts for predicted protein-coding regions, redundancy was reduced by clustering the full set of deduced amino acid sequences using CD-HIT [128,129], with a similarity cut-off of 95%.

Deduced protein sequences were annotated with the software Just_Annotate_My_proteins (JAMp; http://jamps.sourceforge.net/), a workflow that exploits ontologies associated with entries in the Swiss-Prot database (the reviewed subset of the UniProt Knowledgebase/UniProtKB) to annotate new protein sequences based on similarity to high-quality, manually curated proteins present in the Swiss-Prot database. The JAMp workflow has been described previously [42]. Briefly, JAMp uses HHblits [130] to search hidden markov models (HHMs) derived from a set of unknown proteins against HMMs derived from Swiss-Prot database, which provides greater sensitivity than conventional similarity-based searches. Up to 10 HHblits alignments that met similarity criteria (homology probability ≥ 80%; HHblits score ≥ 70; homology e-value ≤ 1e-10; homology p-value 1e-12) were used for functional annotation of predicted rainbowfish proteins. To assign putative functions to predicted proteins, controlled vocabularies associated with matching UniProtKB entries were linked with a rainbowfish sequence only when experimental evidence for functional terms existed (annotations derived from sequence similarity alone were not considered). Controlled vocabularies used for functional annotation included Gene Ontology [46], EggNOG [48], and KEGG [47] and subsets of these databases. Functional annotations, ORFs and transcripts were stored in a PostgreSQL database for access via a web browser-based viewer built with ExtJS4 and canvasXpress.

Additional functional annotations were inferred by sequence similarity to other teleost sequences using BLAST searches [52,131], using controlled vocabularies obtained from the Gene Ontology database via BLAST2GO software [132] or extracted from matching sequences in the NCBI conserved domain database (CDD) using reverse position-specific BLAST [133].

Completeness of the transcriptome was assessed using CEGMA v2.4 [49] (which depends on blast+ v2.2.25, geneid v1.4.4, genewise v2.2.4 and hmmer v3.0) or by sequence similarity searches in amino acid space conducted using BLASTP. For the BLASTP searches, we used *O*. *latipes* CEGs from http://korflab.ucdavis.edu/Datasets/genome_completeness/ and the entire predicted proteome derived from the genome assembly from Ensembl release 76 (ftp://ftp.ensembl.org/pub/release-76/fasta/oryzias_latipes/pep/). Query coverage histograms were prepared with R using the built-in Stats package [134].

Geneious v7 was used to prepare alignments, for phylogenetic analyses, as a front-end for sequence similarity searches and for general curation and review of sequence data.

### Confirmation of ERα transcript variants

Total RNA (1 μg) from brain, liver and gonads from male and female fish was reverse transcribed using the Quantitect® kit (Qiagen Pty. Ltd., Australia) according to the manufacturer’s protocol, except that the incubation time for cDNA synthesis was extended to 1 h. PCR reactions (20 μL) contained 0.4 units Phusion® HotStart II polymerase (Thermo Fisher Scientific, Australia), 1 × HF buffer (Thermo Fisher Scientific, Australia), 0.5 μM dNTP mix (New England Biolabs, Inc.), 0.5 μM each primer and 1 μL of ten-fold diluted cDNA.

End-point PCR was conducted using Phusion HotStart II polymerase with 35–40 cycles. Optimal annealing temperatures for PCR reactions conducted using were calculated as recommended by the manufacturer (www.thermoscientificbio.com/webtools/tmc/). Extension times used were 30 s per kb of the largest expected product. Products were separated on 1.2% agarose gels in TAE buffer and stained post-electrophoresis using GelRed (Biotium, Inc.).

## Supporting Information

S1 Fig Alignment of predicted rainbowfish ERα transcript variants with the Japanese medaka *esr1* gene sequence. (PDF)

S1 Table Data yields for six RNA-Seq libraries generated from male and female rainbowfish tissues and sequenced using two lanes of an Illumina HiSeq 2000 instrument. (DOCX)

S2 Table Putative rainbowfish transcripts and coding sequence identifiers homologous to core eukaryotic genes (CEGs) from the genome of Japanese medaka (*Oryzias latipes*). (CSV)

S3 Table Putative rainbowfish transcripts and coding sequence identifiers homologous to the entire predicted from the genome of Japanese medaka (*Oryzias latipes*). (CSV)

S4 Table Rainbowfish transcripts encoding candidate nuclear receptors identified using conserved domain searches. Transcripts listed contain putative coding sequences matching conserved DNA-binding domains (Pfam cl02596; ‘NR_DBD_like’) and ligand-binding domains (Pfam cl11397; ‘NR_LBD’) from known nuclear receptors.
(CSV)

S5 Table Rainbowfish transcripts encoding candidate nuclear receptors. Transcripts were identified by sequence similarity to predicted nuclear receptors from Japanese medaka (*Oryzias latipes*; obtained from KEGG BRITE ola03310).
(DOCX)

S6 Table Rainbowfish transcripts encoding candidate steroidogenic enzymes. Transcripts were identified by sequence similarity to predicted steroidogenic enzymes from Japanese medaka (*Oryzias latipes*; obtained from KEGG pathway 00140).
(CSV)

S7 Table Rainbowfish transcripts encoding candidate cytochrome P450-family enzymes. Transcripts were identified by sequence similarity with zebrafish CYP450 protein sequences from the Ensembl zebrafish genome assembly (Zv9) annotated as members of PFAM family PF00067 (cytochrome P450).
(CSV)

S8 Table Rainbowfish transcripts encoding proteins with putative aryl hydrocarbon receptor activity. Transcripts were annotated via the JAMp workflow as being associated with Gene Ontology Molecular Function GO: 4874.
(CSV)
