# Supplementary figures and images for: Identification of Putative Nuclear Receptors and Steroidogenic Enzymes in Murray-Darling Rainbowfish (Melanotaenia fluviatilis) Using RNA-Seq and De Novo Transcriptome Assembly
Source: PLoS One. 2015 Nov 23;10(11):e0142636. doi: 10.1371/journal.pone.0142636 (PMC4658143; doi:10.1371/journal.pone.0142636)

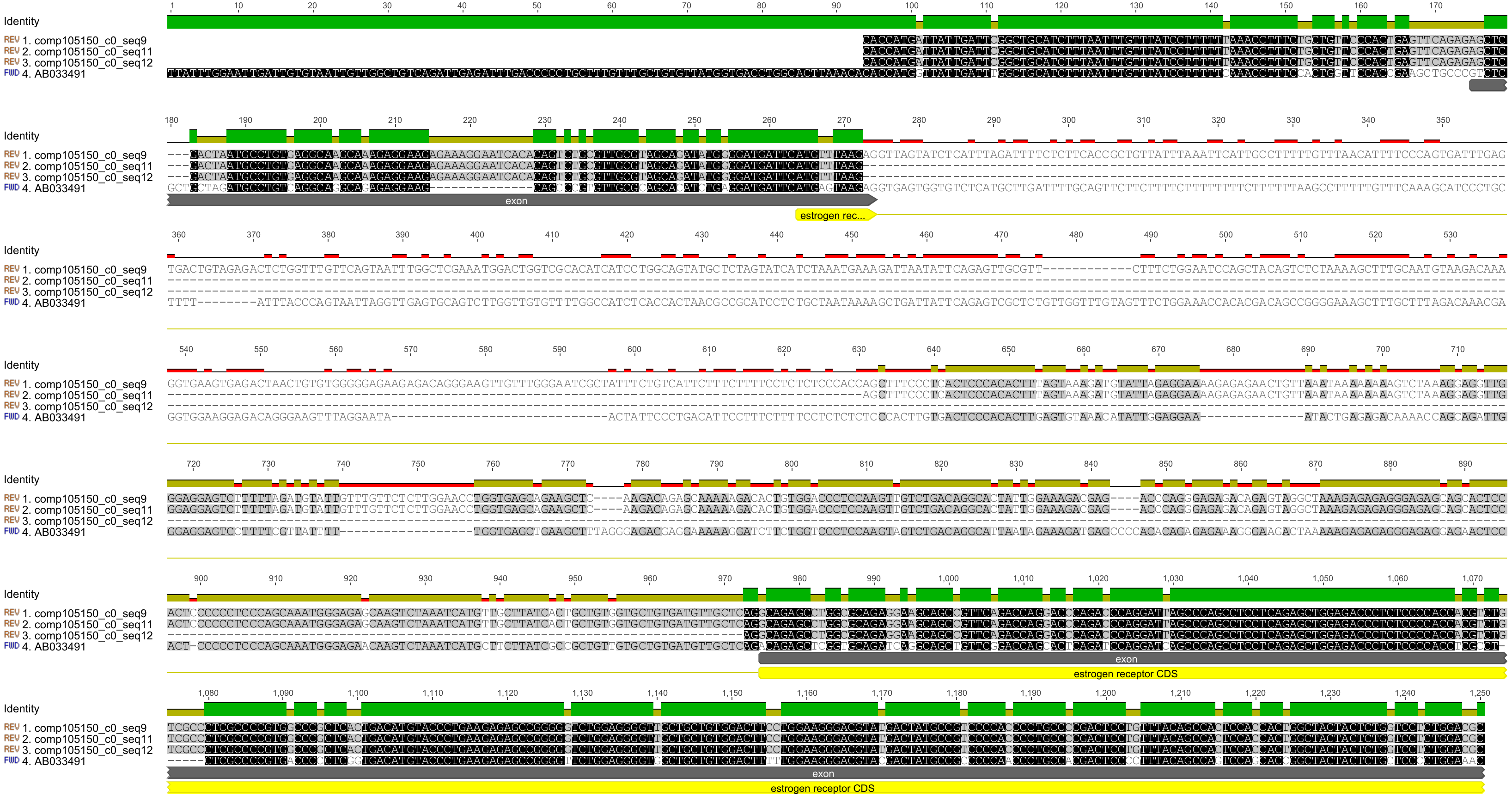

Supplement: S1 Fig — (PDF) [file pone.0142636.s001.pdf]
