# Supplementary material for: Identification of Putative Nuclear Receptors and Steroidogenic Enzymes in Murray-Darling Rainbowfish (Melanotaenia fluviatilis) Using RNA-Seq and De Novo Transcriptome Assembly
Source: PLoS One. 2015 Nov 23;10(11):e0142636. doi: 10.1371/journal.pone.0142636 (PMC4658143; doi:10.1371/journal.pone.0142636)
Supplement: S1 Table — (DOCX) [file pone.0142636.s002.docx]

**Table S1.** Data yields for each male and female tissues resulting from sequencing of six libraries using two lanes of Illumina HiSeq 2000.

| Sample | Paired Reads | Data Yield (bases) |
| --- | --- | --- |
| Female liver | 37,015,957 | 7.40 Gb |
| Female brain | 52,862,830 | 10.57 Gb |
| Ovaries | 30,490,268 | 6.10 Gb |
| Male liver | 41,109,506 | 8.22 Gb |
| Male brain | 44,802,240 | 8.96 Gb |
| Testes | 52,625,459 | 10.53 Gb |
| Total | 258,906,260 | 51.78 Gb |
