# Supplementary material for: Identification of Putative Nuclear Receptors and Steroidogenic Enzymes in Murray-Darling Rainbowfish (Melanotaenia fluviatilis) Using RNA-Seq and De Novo Transcriptome Assembly
Source: PLoS One. 2015 Nov 23;10(11):e0142636. doi: 10.1371/journal.pone.0142636 (PMC4658143; doi:10.1371/journal.pone.0142636)
Supplement: S5 Table — Transcripts were identified by sequence similarity to predicted nuclear receptors from Japanese medaka (Oryzias latipes; obtained from KEGG BRITE ola03310). (DOCX) [file pone.0142636.s006.docx]

| Rainbowfish transcript\|CDS identifier | Medaka query (GenBank accession) | Query description  (abbreviation) | E-Value | Bit-Score | Query coverage | % Identical sites | Query end | Query start | Hit start | Hit end |
| --- | --- | --- | --- | --- | --- | --- | --- | --- | --- | --- |
| cds.comp43664_c0_seq1\|m.2708 comp43664_c0_seq1\|g.2708 ORF comp43664_c0_seq1\|g.2708 comp43664_c0_seq1\|m.2708 type:complete len:293 (+) comp43664_c0_seq1:198-1076(+) | gi\|160707939\|ref\|NP_001104259.1\| | orphan nuclear receptor Dax-1 [Oryzias latipes]  (DAX-1) | 6.76E-131 | 465.307 | 100.00% | 80.20% | 287 | 1 | 1 | 292 |
| cds.comp63744_c0_seq1\|m.5886 comp63744_c0_seq1\|g.5886 ORF comp63744_c0_seq1\|g.5886 comp63744_c0_seq1\|m.5886 type:complete len:576 (-) comp63744_c0_seq1:1286-3013(-) | gi\|432866253\|ref\|XP_004070760.1\| | PREDICTED: nuclear receptor subfamily 1 group D member 2-like [Oryzias latipes]  (NR1D2) | 0 | 941.799 | 100.00% | 86.30% | 574 | 1 | 1 | 575 |
| cds.comp68246_c0_seq1\|m.6870 comp68246_c0_seq1\|g.6870 ORF comp68246_c0_seq1\|g.6870 comp68246_c0_seq1\|m.6870 type:complete len:451 (-) comp68246_c0_seq1:393-1745(-) | gi\|432889352\|ref\|XP_004075234.1\| | PREDICTED: estrogen-related receptor gamma-like [Oryzias latipes]  (ERRγ) | 2.54E-86 | 317.775 | 64.86% | 58.30% | 365 | 102 | 113 | 381 |
| cds.comp68246_c0_seq1\|m.6870 comp68246_c0_seq1\|g.6870 ORF comp68246_c0_seq1\|g.6870 comp68246_c0_seq1\|m.6870 type:complete len:451 (-) comp68246_c0_seq1:393-1745(-) | gi\|302129672\|ref\|NP_001098389.2\| | estrogen-related receptor gamma type 1 [Oryzias latipes]  (ERRγ1) | 0 | 724.161 | 100.00% | 79.20% | 445 | 1 | 15 | 450 |
| cds.comp68246_c0_seq1\|m.6870 comp68246_c0_seq1\|g.6870 ORF comp68246_c0_seq1\|g.6870 comp68246_c0_seq1\|m.6870 type:complete len:451 (-) comp68246_c0_seq1:393-1745(-) | gi\|251823756\|ref\|NP_001156563.1\| | estrogen-related receptor beta type 2 [Oryzias latipes]  (ERRβ2) | 0 | 754.207 | 100.00% | 82.20% | 431 | 1 | 15 | 450 |
| cds.comp68246_c0_seq1\|m.6870 comp68246_c0_seq1\|g.6870 ORF comp68246_c0_seq1\|g.6870 comp68246_c0_seq1\|m.6870 type:complete len:451 (-) comp68246_c0_seq1:393-1745(-) | gi\|157278575\|ref\|NP_001098388.1\| | estrogen-related receptor beta type 1 [Oryzias latipes]  (ERRβ1) | 0 | 880.552 | 100.00% | 97.50% | 433 | 1 | 15 | 450 |
| cds.comp79466_c0_seq1\|m.10100 comp79466_c0_seq1\|g.10100 ORF comp79466_c0_seq1\|g.10100 comp79466_c0_seq1\|m.10100 type:complete len:506 (+) comp79466_c0_seq1:398-1915(+) | gi\|523704577\|ref\|NP_001265836.1\| | peroxisome proliferator activated receptor beta [Oryzias latipes]  (PPARβ) | 0 | 931.398 | 100.00% | 89.70% | 506 | 1 | 1 | 505 |
| cds.comp79717_c0_seq1\|m.10289 comp79717_c0_seq1\|g.10289 ORF comp79717_c0_seq1\|g.10289 comp79717_c0_seq1\|m.10289 type:5prime_partial len:271 (-) comp79717_c0_seq1:345-1157(-) | gi\|432861654\|ref\|XP_004069672.1\| | PREDICTED: photoreceptor-specific nuclear receptor-like [Oryzias latipes]  (PNR) | 2.63E-101 | 367.851 | 59.13% | 74.20% | 416 | 171 | 23 | 270 |
| cds.comp79717_c0_seq1\|m.10289 comp79717_c0_seq1\|g.10289 ORF comp79717_c0_seq1\|g.10289 comp79717_c0_seq1\|m.10289 type:5prime_partial len:271 (-) comp79717_c0_seq1:345-1157(-) | gi\|432850560\|ref\|XP_004066811.1\| | PREDICTED: hepatocyte nuclear factor 4-gamma-like [Oryzias latipes]  (HNF4γ) | 3.30E-99 | 360.533 | 47.27% | 98.90% | 385 | 204 | 89 | 270 |
| cds.comp79717_c0_seq1\|m.10289 comp79717_c0_seq1\|g.10289 ORF comp79717_c0_seq1\|g.10289 comp79717_c0_seq1\|m.10289 type:5prime_partial len:271 (-) comp79717_c0_seq1:345-1157(-) | gi\|432850556\|ref\|XP_004066809.1\| | PREDICTED: photoreceptor-specific nuclear receptor-like [Oryzias latipes]  (PNR) | 1.37E-147 | 521.546 | 62.65% | 97.00% | 431 | 162 | 1 | 270 |
| cds.comp82092_c0_seq1\|m.11717 comp82092_c0_seq1\|g.11717 ORF comp82092_c0_seq1\|g.11717 comp82092_c0_seq1\|m.11717 type:complete len:259 (-) comp82092_c0_seq1:377-1153(-) | gi\|432907930\|ref\|XP_004077711.1\| | PREDICTED: nuclear receptor subfamily 0 group B member 2-like [Oryzias latipes]  (NR0B2) | 2.02E-128 | 456.833 | 92.14% | 84.50% | 280 | 23 | 1 | 258 |
| cds.comp82210_c1_seq3\|m.11801 comp82210_c1_seq3\|g.11801 ORF comp82210_c1_seq3\|g.11801 comp82210_c1_seq3\|m.11801 type:complete len:630 (+) comp82210_c1_seq3:168-2057(+) | gi\|432866384\|ref\|XP_004070824.1\| | PREDICTED: LOW QUALITY PROTEIN: nuclear receptor subfamily 2 group C member 2-like [Oryzias latipes]  (NR2C2) | 0 | 1070.07 | 100.00% | 84.70% | 609 | 1 | 1 | 629 |
| cds.comp83454_c0_seq1\|m.12530 comp83454_c0_seq1\|g.12530 ORF comp83454_c0_seq1\|g.12530 comp83454_c0_seq1\|m.12530 type:complete len:445 (-) comp83454_c0_seq1:1019-2353(-) | gi\|432957376\|ref\|XP_004085823.1\| | "PREDICTED: nuclear receptor subfamily 1 group D member 2-like, partial [Oryzias latipes]"  (NR1D2) | 1.60E-163 | 574.704 | 80.11% | 70.20% | 528 | 106 | 1 | 444 |
| cds.comp83657_c0_seq1\|m.12811 comp83657_c0_seq1\|g.12811 ORF comp83657_c0_seq1\|g.12811 comp83657_c0_seq1\|m.12811 type:complete len:464 (-) comp83657_c0_seq1:3307-4698(-) | gi\|432851594\|ref\|XP_004066988.1\| | PREDICTED: oxysterols receptor LXR-alpha-like [Oryzias latipes]  (LXRα) | 0 | 748.814 | 95.37% | 97.30% | 389 | 19 | 94 | 463 |
| cds.comp83709_c0_seq2\|m.12880 comp83709_c0_seq2\|g.12880 ORF comp83709_c0_seq2\|g.12880 comp83709_c0_seq2\|m.12880 type:complete len:466 (-) comp83709_c0_seq2:586-1983(-) | gi\|432867223\|ref\|XP_004071086.1\| | PREDICTED: hepatocyte nuclear factor 4-alpha-like [Oryzias latipes]  (HNF4α) | 0 | 815.068 | 98.61% | 87.90% | 431 | 7 | 2 | 465 |
| cds.comp84494_c0_seq1\|m.14114 comp84494_c0_seq1\|g.14114 ORF comp84494_c0_seq1\|g.14114 comp84494_c0_seq1\|m.14114 type:complete len:329 (-) comp84494_c0_seq1:148-1134(-) | gi\|432935701\|ref\|XP_004082046.1\| | PREDICTED: nuclear receptor subfamily 1 group I member 2 [Oryzias latipes]  (NR1I2) | 8.70E-115 | 412.535 | 76.44% | 61.10% | 433 | 103 | 1 | 328 |
| cds.comp86668_c3_seq1\|m.16516 comp86668_c3_seq1\|g.16516 ORF comp86668_c3_seq1\|g.16516 comp86668_c3_seq1\|m.16516 type:5prime_partial len:327 (+) comp86668_c3_seq1:3-983(+) | gi\|157278393\|ref\|NP_001098298.1\| | LRH-1 [Oryzias latipes]  (liver receptor homolog-1; LRH-1) | 0 | 673.315 | 62.33% | 98.80% | 523 | 198 | 1 | 326 |
| cds.comp89666_c1_seq1\|m.20492 comp89666_c1_seq1\|g.20492 ORF comp89666_c1_seq1\|g.20492 comp89666_c1_seq1\|m.20492 type:complete len:426 (-) comp89666_c1_seq1:1070-2347(-) | gi\|190570210\|ref\|NP_001121989.1\| | vitamin D receptor beta [Oryzias latipes]  (VDRβ) | 0 | 797.734 | 100.00% | 92.70% | 425 | 1 | 1 | 425 |
| cds.comp89666_c1_seq1\|m.20492 comp89666_c1_seq1\|g.20492 ORF comp89666_c1_seq1\|g.20492 comp89666_c1_seq1\|m.20492 type:complete len:426 (-) comp89666_c1_seq1:1070-2347(-) | gi\|190570208\|ref\|NP_001121988.1\| | vitamin D receptor alpha [Oryzias latipes]  (VDRα) | 0 | 774.622 | 100.00% | 88.20% | 420 | 1 | 1 | 425 |
| cds.comp89740_c0_seq1\|m.20594 comp89740_c0_seq1\|g.20594 ORF comp89740_c0_seq1\|g.20594 comp89740_c0_seq1\|m.20594 type:5prime_partial len:366 (+) comp89740_c0_seq1:2-1099(+) | gi\|432909380\|ref\|XP_004078181.1\| | PREDICTED: nuclear receptor ROR-alpha-like [Oryzias latipes]  (RORα) | 8.57E-180 | 628.632 | 74.55% | 82.70% | 495 | 127 | 10 | 365 |
| cds.comp91472_c0_seq1\|m.24241 comp91472_c0_seq1\|g.24241 ORF comp91472_c0_seq1\|g.24241 comp91472_c0_seq1\|m.24241 type:3prime_partial len:255 (-) comp91472_c0_seq1:1-762(-) | gi\|432871968\|ref\|XP_004072051.1\| | PREDICTED: thyroid hormone receptor alpha-like [Oryzias latipes]  (TRα) | 8.06E-112 | 402.519 | 53.58% | 93.10% | 243 | 27 | 38 | 254 |
| cds.comp91472_c0_seq2\|m.24243 comp91472_c0_seq2\|g.24243 ORF comp91472_c0_seq2\|g.24243 comp91472_c0_seq2\|m.24243 type:3prime_partial len:248 (-) comp91472_c0_seq2:1-741(-) | gi\|157278151\|ref\|NP_001098175.1\| | thyroid hormone receptor alpha [Oryzias latipes]  (TRα) | 2.41E-115 | 414.461 | 60.58% | 84.30% | 249 | 1 | 1 | 247 |
| cds.comp92726_c0_seq1\|m.27858 comp92726_c0_seq1\|g.27858 ORF comp92726_c0_seq1\|g.27858 comp92726_c0_seq1\|m.27858 type:5prime_partial len:388 (+) comp92726_c0_seq1:1-1164(+) | gi\|432929881\|ref\|XP_004081273.1\| | PREDICTED: hepatocyte nuclear factor 4-alpha-like [Oryzias latipes]  (HNF4α) | 0 | 726.087 | 85.24% | 92.20% | 454 | 68 | 1 | 387 |
| cds.comp92755_c1_seq3\|m.27963 comp92755_c1_seq3\|g.27963 ORF comp92755_c1_seq3\|g.27963 comp92755_c1_seq3\|m.27963 type:3prime_partial len:313 (-) comp92755_c1_seq3:1-936(-) | gi\|432913184\|ref\|XP_004078947.1\| | PREDICTED: nuclear receptor subfamily 2 group F member 6-like [Oryzias latipes]  (NR2F6) | 2.34E-168 | 590.497 | 76.11% | 92.60% | 309 | 1 | 1 | 312 |
| cds.comp93992_c0_seq1\|m.32225 comp93992_c0_seq1\|g.32225 ORF comp93992_c0_seq1\|g.32225 comp93992_c0_seq1\|m.32225 type:complete len:590 (+) comp93992_c0_seq1:151-1920(+) | gi\|432866776\|ref\|XP_004070930.1\| | PREDICTED: nuclear receptor subfamily 4 group A member 1-like [Oryzias latipes]  (NR4A1) | 0 | 696.427 | 100.00% | 62.50% | 571 | 1 | 1 | 589 |
| cds.comp93992_c0_seq1\|m.32225 comp93992_c0_seq1\|g.32225 ORF comp93992_c0_seq1\|g.32225 comp93992_c0_seq1\|m.32225 type:complete len:590 (+) comp93992_c0_seq1:151-1920(+) | gi\|432859925\|ref\|XP_004069304.1\| | PREDICTED: nuclear receptor subfamily 4 group A member 1-like [Oryzias latipes]  (NR4A1) | 0 | 941.028 | 100.00% | 81.20% | 585 | 1 | 1 | 589 |
| cds.comp94434_c0_seq2\|m.33968 comp94434_c0_seq2\|g.33968 ORF comp94434_c0_seq2\|g.33968 comp94434_c0_seq2\|m.33968 type:complete len:325 (-) comp94434_c0_seq2:349-1323(-) | gi\|432848602\|ref\|XP_004066427.1\| | PREDICTED: nuclear receptor subfamily 4 group A member 2-like [Oryzias latipes]  (NR4A2) | 4.82E-157 | 553.132 | 63.15% | 87.90% | 483 | 179 | 18 | 324 |
| cds.comp95426_c0_seq4\|m.38710 comp95426_c0_seq4\|g.38710 ORF comp95426_c0_seq4\|g.38710 comp95426_c0_seq4\|m.38710 type:complete len:448 (+) comp95426_c0_seq4:44-1387(+) | gi\|432889046\|ref\|XP_004075118.1\| | PREDICTED: nuclear receptor subfamily 5 group A member 2-like [Oryzias latipes]  (NR5A2) | 1.99E-140 | 497.664 | 99.56% | 58.80% | 451 | 1 | 1 | 445 |
| cds.comp95556_c0_seq1\|m.39498 comp95556_c0_seq1\|g.39498 ORF comp95556_c0_seq1\|g.39498 comp95556_c0_seq1\|m.39498 type:complete len:462 (+) comp95556_c0_seq1:287-1672(+) | gi\|251823758\|ref\|NP_001156564.1\| | estrogen-related receptor gamma type 2 [Oryzias latipes]  (ERRγ2) | 0 | 793.882 | 100.00% | 91.40% | 439 | 1 | 24 | 461 |
| cds.comp96203_c0_seq1\|m.42360 comp96203_c0_seq1\|g.42360 ORF comp96203_c0_seq1\|g.42360 comp96203_c0_seq1\|m.42360 type:complete len:535 (-) comp96203_c0_seq1:1286-2890(-) | gi\|259013197\|ref\|NP_001158348.1\| | peroxisome proliferator-activated receptor gamma [Oryzias latipes]  (PPARγ) | 0 | 914.064 | 100.00% | 86.40% | 531 | 1 | 1 | 534 |
| cds.comp96886_c0_seq4\|m.46654 comp96886_c0_seq4\|g.46654 ORF comp96886_c0_seq4\|g.46654 comp96886_c0_seq4\|m.46654 type:complete len:609 (+) comp96886_c0_seq4:339-2165(+) | gi\|432910618\|ref\|XP_004078441.1\| | PREDICTED: nuclear receptor subfamily 1 group D member 2-like [Oryzias latipes]  (NR1D2) | 0 | 824.698 | 100.00% | 77.00% | 566 | 1 | 1 | 608 |
| cds.comp97238_c0_seq1\|m.48939 comp97238_c0_seq1\|g.48939 ORF comp97238_c0_seq1\|g.48939 comp97238_c0_seq1\|m.48939 type:5prime_partial len:385 (+) comp97238_c0_seq1:2-1156(+) | gi\|157278145\|ref\|NP_001098172.1\| | estrogen receptor beta [Oryzias latipes]  (ERβ) | 0 | 644.81 | 68.33% | 81.80% | 562 | 179 | 1 | 384 |
| cds.comp98381_c2_seq1\|m.58695 comp98381_c2_seq1\|g.58695 ORF comp98381_c2_seq1\|g.58695 comp98381_c2_seq1\|m.58695 type:3prime_partial len:720 (+) comp98381_c2_seq1:337-2493(+) | gi\|253314476\|ref\|NP_001156605.1\| | glucocorticoid receptor [Oryzias latipes]  (GR) | 0 | 1203.35 | 92.28% | 82.50% | 717 | 1 | 1 | 719 |
| cds.comp98578_c0_seq1\|m.60686 comp98578_c0_seq1\|g.60686 ORF comp98578_c0_seq1\|g.60686 comp98578_c0_seq1\|m.60686 type:5prime_partial len:448 (-) comp98578_c0_seq1:3172-4515(-) | gi\|432853451\|ref\|XP_004067713.1\| | PREDICTED: nuclear receptor ROR-beta-like [Oryzias latipes]  (RAR-related orphan receptor beta; RORβ) | 0 | 810.446 | 94.29% | 87.70% | 473 | 28 | 1 | 447 |
| cds.comp98731_c0_seq1\|m.61974 comp98731_c0_seq1\|g.61974 ORF comp98731_c0_seq1\|g.61974 comp98731_c0_seq1\|m.61974 type:complete len:470 (+) comp98731_c0_seq1:238-1647(+) | gi\|432910610\|ref\|XP_004078439.1\| | PREDICTED: retinoic acid receptor beta-like [Oryzias latipes]  (RARβ) | 0 | 783.867 | 99.08% | 92.30% | 429 | 1 | 26 | 456 |
| cds.comp98731_c0_seq1\|m.61974 comp98731_c0_seq1\|g.61974 ORF comp98731_c0_seq1\|g.61974 comp98731_c0_seq1\|m.61974 type:complete len:470 (+) comp98731_c0_seq1:238-1647(+) | gi\|432883778\|ref\|XP_004074348.1\| | PREDICTED: retinoic acid receptor beta [Oryzias latipes]  (RARβ) | 0 | 634.024 | 90.65% | 74.80% | 417 | 1 | 26 | 456 |
| cds.comp98731_c0_seq1\|m.61974 comp98731_c0_seq1\|g.61974 ORF comp98731_c0_seq1\|g.61974 comp98731_c0_seq1\|m.61974 type:complete len:470 (+) comp98731_c0_seq1:238-1647(+) | gi\|432865288\|ref\|XP_004070509.1\| | PREDICTED: retinoic acid receptor gamma-A isoform 2 [Oryzias latipes]  (RARγ-2) | 1.04E-166 | 585.489 | 66.67% | 79.20% | 419 | 80 | 109 | 449 |
| cds.comp98731_c0_seq1\|m.61974 comp98731_c0_seq1\|g.61974 ORF comp98731_c0_seq1\|g.61974 comp98731_c0_seq1\|m.61974 type:complete len:470 (+) comp98731_c0_seq1:238-1647(+) | gi\|432865286\|ref\|XP_004070508.1\| | PREDICTED: retinoic acid receptor gamma-A isoform 1 [Oryzias latipes]  (RARγ-1) | 1.05E-168 | 592.038 | 81.87% | 70.50% | 411 | 1 | 26 | 449 |
| cds.comp98731_c0_seq1\|m.61974 comp98731_c0_seq1\|g.61974 ORF comp98731_c0_seq1\|g.61974 comp98731_c0_seq1\|m.61974 type:complete len:470 (+) comp98731_c0_seq1:238-1647(+) | gi\|432859604\|ref\|XP_004069176.1\| | PREDICTED: retinoic acid receptor gamma-A isoform 2 [Oryzias latipes]  (RARγ-2) | 1.19E-165 | 581.637 | 69.98% | 77.50% | 424 | 80 | 109 | 454 |
| cds.comp98731_c0_seq1\|m.61974 comp98731_c0_seq1\|g.61974 ORF comp98731_c0_seq1\|g.61974 comp98731_c0_seq1\|m.61974 type:complete len:470 (+) comp98731_c0_seq1:238-1647(+) | gi\|432859602\|ref\|XP_004069175.1\| | PREDICTED: retinoic acid receptor gamma-A isoform 1 [Oryzias latipes]  (RARγ-2) | 3.43E-166 | 583.563 | 85.77% | 68.80% | 416 | 1 | 26 | 454 |
| cds.comp98865_c5_seq2\|m.63275 comp98865_c5_seq2\|g.63275 ORF comp98865_c5_seq2\|g.63275 comp98865_c5_seq2\|m.63275 type:5prime_partial len:351 (-) comp98865_c5_seq2:513-1565(-) | gi\|432921789\|ref\|XP_004080224.1\| | PREDICTED: retinoic acid receptor alpha-A [Oryzias latipes]  RARα-A | 0 | 640.573 | 77.04% | 98.30% | 453 | 105 | 1 | 350 |
| cds.comp100164_c1_seq1\|m.79221 comp100164_c1_seq1\|g.79221 ORF comp100164_c1_seq1\|g.79221 comp100164_c1_seq1\|m.79221 type:complete len:766 (+) comp100164_c1_seq1:280-2577(+) | gi\|157278104\|ref\|NP_001098151.1\| | androgen receptor beta [Oryzias latipes]  (ARβ) | 0 | 1162.9 | 100.00% | 75.00% | 744 | 1 | 1 | 765 |
| cds.comp100355_c1_seq1\|m.82115 comp100355_c1_seq1\|g.82115 ORF comp100355_c1_seq1\|g.82115 comp100355_c1_seq1\|m.82115 type:complete len:996 (+) comp100355_c1_seq1:409-3396(+) | gi\|253314470\|ref\|NP_001156601.1\| | mineralocorticoid receptor [Oryzias latipes]  (MR) | 0 | 1781.53 | 100.00% | 88.20% | 994 | 1 | 1 | 995 |
| cds.comp100560_c2_seq1\|m.84660 comp100560_c2_seq1\|g.84660 ORF comp100560_c2_seq1\|g.84660 comp100560_c2_seq1\|m.84660 type:complete len:412 (+) comp100560_c2_seq1:840-2075(+) | gi\|432910588\|ref\|XP_004078428.1\| | PREDICTED: nuclear receptor subfamily 2 group F member 5-like [Oryzias latipes]  (NR2F5) | 7.31E-179 | 625.165 | 95.54% | 76.00% | 386 | 1 | 1 | 408 |
| cds.comp100761_c0_seq4\|m.87191 comp100761_c0_seq4\|g.87191 ORF comp100761_c0_seq4\|g.87191 comp100761_c0_seq4\|m.87191 type:complete len:673 (-) comp100761_c0_seq4:5144-7162(-) | gi\|285026398\|ref\|NP_001165515.1\| | progesterone receptor [Oryzias latipes]  (PR) | 0 | 1024.23 | 100.00% | 74.60% | 628 | 1 | 1 | 672 |
| cds.comp100793_c0_seq16\|m.87923 comp100793_c0_seq16\|g.87923 ORF comp100793_c0_seq16\|g.87923 comp100793_c0_seq16\|m.87923 type:complete len:491 (+) comp100793_c0_seq16:265-1737(+) | gi\|432864386\|ref\|XP_004070296.1\| | PREDICTED: bile acid receptor-like [Oryzias latipes]  (BAR) | 7.64E-171 | 598.971 | 94.71% | 68.80% | 471 | 24 | 34 | 489 |
| cds.comp101322_c2_seq1\|m.97989 comp101322_c2_seq1\|g.97989 ORF comp101322_c2_seq1\|g.97989 comp101322_c2_seq1\|m.97989 type:complete len:427 (+) comp101322_c2_seq1:6409-7689(+) | gi\|432908547\|ref\|XP_004077915.1\| | PREDICTED: nuclear receptor subfamily 4 group A member 3-like isoform 2 [Oryzias latipes]  (NR4A3-like-1) | 0 | 672.159 | 66.95% | 92.50% | 596 | 198 | 35 | 426 |
| cds.comp101322_c2_seq1\|m.97989 comp101322_c2_seq1\|g.97989 ORF comp101322_c2_seq1\|g.97989 comp101322_c2_seq1\|m.97989 type:complete len:427 (+) comp101322_c2_seq1:6409-7689(+) | gi\|432908545\|ref\|XP_004077914.1\| | PREDICTED: nuclear receptor subfamily 4 group A member 3-like isoform 1 [Oryzias latipes]  (NR4A3-like-2) | 0 | 687.567 | 66.61% | 94.90% | 590 | 198 | 35 | 426 |
| cds.comp101322_c2_seq2\|m.97994 comp101322_c2_seq2\|g.97994 ORF comp101322_c2_seq2\|g.97994 comp101322_c2_seq2\|m.97994 type:5prime_partial len:421 (+) comp101322_c2_seq2:2-1264(+) | gi\|432876056\|ref\|XP_004072955.1\| | PREDICTED: nuclear receptor subfamily 6 group A member 1-A-like [Oryzias latipes]  (NR6A1A) | 3.54E-165 | 579.326 | 89.12% | 93.90% | 326 | 32 | 1 | 295 |
| cds.comp101328_c0_seq5\|m.98210 comp101328_c0_seq5\|g.98210 ORF comp101328_c0_seq5\|g.98210 comp101328_c0_seq5\|m.98210 type:complete len:486 (-) comp101328_c0_seq5:327-1784(-) | gi\|432862253\|ref\|XP_004069763.1\| | PREDICTED: bile acid receptor-like [Oryzias latipes]  (BAR) | 0 | 922.539 | 100.00% | 89.50% | 485 | 1 | 1 | 485 |
| cds.comp102185_c0_seq3\|m.112122 comp102185_c0_seq3\|g.112122 ORF comp102185_c0_seq3\|g.112122 comp102185_c0_seq3\|m.112122 type:complete len:434 (+) comp102185_c0_seq3:143-1444(+) | gi\|157278573\|ref\|NP_001098387.1\| | estrogen-related receptor alpha [Oryzias latipes]  (ERRα) | 0 | 771.541 | 100.00% | 97.00% | 433 | 1 | 1 | 433 |
| cds.comp102838_c0_seq1\|m.127413 comp102838_c0_seq1\|g.127413 ORF comp102838_c0_seq1\|g.127413 comp102838_c0_seq1\|m.127413 type:complete len:375 (+) comp102838_c0_seq1:478-1602(+) | gi\|157278483\|ref\|NP_001098343.1\| | nuclear receptor subfamily 2 group E member 1 [Oryzias latipes]  (NR2E1) | 0 | 754.207 | 94.44% | 96.80% | 374 | 1 | 1 | 374 |
| cds.comp102912_c0_seq8\|m.129341 comp102912_c0_seq8\|g.129341 ORF comp102912_c0_seq8\|g.129341 comp102912_c0_seq8\|m.129341 type:complete len:611 (-) comp102912_c0_seq8:2239-4071(-) | gi\|432861275\|ref\|XP_004069587.1\| | PREDICTED: nuclear receptor subfamily 2 group C member 1-like [Oryzias latipes]  (NR2C1) | 0 | 1147.88 | 100.00% | 90.70% | 610 | 1 | 1 | 610 |
| cds.comp102970_c0_seq7\|m.130785 comp102970_c0_seq7\|g.130785 ORF comp102970_c0_seq7\|g.130785 comp102970_c0_seq7\|m.130785 type:complete len:483 (-) comp102970_c0_seq7:1703-3151(-) | gi\|283046716\|ref\|NP_001164304.1\| | androgen receptor alpha subtype [Oryzias latipes]  (ARα) | 0 | 743.036 | 57.21% | 91.10% | 687 | 295 | 89 | 482 |
| cds.comp102970_c0_seq7\|m.130785 comp102970_c0_seq7\|g.130785 ORF comp102970_c0_seq7\|g.130785 comp102970_c0_seq7\|m.130785 type:complete len:483 (-) comp102970_c0_seq7:1703-3151(-) | gi\|171544933\|ref\|NP_001116383.1\| | androgen receptor beta [Oryzias latipes]  (ARβ) | 0 | 739.954 | 57.33% | 90.90% | 689 | 295 | 89 | 482 |
| cds.comp102970_c0_seq9\|m.130789 comp102970_c0_seq9\|g.130789 ORF comp102970_c0_seq9\|g.130789 comp102970_c0_seq9\|m.130789 type:complete len:788 (-) comp102970_c0_seq9:928-3291(-) | gi\|432895815\|ref\|XP_004076175.1\| | PREDICTED: glucocorticoid receptor-like [Oryzias latipes]  (GR) | 0 | 1115.14 | 91.44% | 73.60% | 780 | 1 | 1 | 777 |
| cds.comp103241_c1_seq2\|m.137157 comp103241_c1_seq2\|g.137157 ORF comp103241_c1_seq2\|g.137157 comp103241_c1_seq2\|m.137157 type:complete len:487 (+) comp103241_c1_seq2:221-1681(+) | gi\|157278133\|ref\|NP_001098166.1\| | FTZ-F1 [Oryzias latipes]  (Fushi tarazu-F1; FTZ-F1) | 0 | 927.546 | 100.00% | 95.70% | 486 | 1 | 1 | 486 |
| cds.comp103362_c3_seq1\|m.140216 comp103362_c3_seq1\|g.140216 ORF comp103362_c3_seq1\|g.140216 comp103362_c3_seq1\|m.140216 type:complete len:473 (+) comp103362_c3_seq1:327-1745(+) | gi\|432874724\|ref\|XP_004072561.1\| | PREDICTED: nuclear receptor ROR-beta-like [Oryzias latipes]  (RORβ) | 0 | 979.163 | 100.00% | 98.10% | 472 | 1 | 1 | 472 |
| cds.comp103362_c3_seq2\|m.140218 comp103362_c3_seq2\|g.140218 ORF comp103362_c3_seq2\|g.140218 comp103362_c3_seq2\|m.140218 type:5prime_partial len:498 (+) comp103362_c3_seq2:1-1494(+) | gi\|432861682\|ref\|XP_004069686.1\| | PREDICTED: nuclear receptor ROR-alpha-like [Oryzias latipes]  (RORα) | 0 | 957.592 | 88.25% | 99.30% | 519 | 62 | 40 | 497 |
| cds.comp103476_c2_seq6\|m.142553 comp103476_c2_seq6\|g.142553 ORF comp103476_c2_seq6\|g.142553 comp103476_c2_seq6\|m.142553 type:complete len:425 (-) comp103476_c2_seq6:3606-4880(-) | gi\|190570200\|ref\|NP_001121984.1\| | estrogen receptor beta 2 [Oryzias latipes]  (ERβ2) | 2.95E-157 | 553.903 | 60.52% | 87.50% | 524 | 197 | 1 | 325 |
| cds.comp103883_c2_seq1\|m.152848 comp103883_c2_seq1\|g.152848 ORF comp103883_c2_seq1\|g.152848 comp103883_c2_seq1\|m.152848 type:complete len:445 (-) comp103883_c2_seq1:2051-3385(-) | gi\|432908440\|ref\|XP_004077862.1\| | PREDICTED: retinoic acid receptor RXR-beta-A [Oryzias latipes]  (RXR-βA) | 0 | 797.349 | 100.00% | 92.60% | 443 | 1 | 1 | 444 |
| cds.comp103883_c2_seq7\|m.152854 comp103883_c2_seq7\|g.152854 ORF comp103883_c2_seq7\|g.152854 comp103883_c2_seq7\|m.152854 type:complete len:463 (-) comp103883_c2_seq7:725-2113(-) | gi\|432883656\|ref\|XP_004074314.1\| | PREDICTED: retinoic acid receptor RXR-beta-A [Oryzias latipes]  (RXRβ-A) | 0 | 880.937 | 95.70% | 96.20% | 465 | 21 | 21 | 462 |
| cds.comp103883_c2_seq12\|m.152869 comp103883_c2_seq12\|g.152869 ORF comp103883_c2_seq12\|g.152869 comp103883_c2_seq12\|m.152869 type:complete len:451 (-) comp103883_c2_seq12:512-1864(-) | gi\|432856175\|ref\|XP_004068390.1\| | PREDICTED: retinoic acid receptor RXR-gamma-B [Oryzias latipes]  (RXRγB) | 0 | 720.694 | 95.16% | 81.90% | 455 | 23 | 26 | 450 |
| cds.comp103926_c0_seq4\|m.153805 comp103926_c0_seq4\|g.153805 ORF comp103926_c0_seq4\|g.153805 comp103926_c0_seq4\|m.153805 type:complete len:486 (+) comp103926_c0_seq4:240-1697(+) | gi\|259013193\|ref\|NP_001158347.1\| | peroxisome proliferator-activated receptor alpha 2 [Oryzias latipes]  (PPARα-2 | 0 | 811.216 | 100.00% | 82.10% | 480 | 1 | 9 | 485 |
| cds.comp103949_c2_seq5\|m.154602 comp103949_c2_seq5\|g.154602 ORF comp103949_c2_seq5\|g.154602 comp103949_c2_seq5\|m.154602 type:complete len:229 (-) comp103949_c2_seq5:2871-3557(-) | gi\|157278123\|ref\|NP_001098160.1\| | thyroid hormone receptor beta [Oryzias latipes]  (TRβ) | 1.45E-103 | 375.17 | 54.23% | 90.20% | 206 | 2 | 19 | 223 |
| cds.comp104204_c10_seq1\|m.161520 comp104204_c10_seq1\|g.161520 ORF comp104204_c10_seq1\|g.161520 comp104204_c10_seq1\|m.161520 type:complete len:469 (+) comp104204_c10_seq1:183-1589(+) | gi\|432862598\|ref\|XP_004069934.1\| | PREDICTED: peroxisome proliferator-activated receptor alpha [Oryzias latipes]  PPARα | 0 | 773.081 | 97.09% | 93.30% | 412 | 13 | 70 | 468 |
| cds.comp104218_c0_seq1\|m.161918 comp104218_c0_seq1\|g.161918 ORF comp104218_c0_seq1\|g.161918 comp104218_c0_seq1\|m.161918 type:complete len:424 (-) comp104218_c0_seq1:105-1376(-) | gi\|432861727\|ref\|XP_004069708.1\| | PREDICTED: COUP transcription factor 2-like isoform 2 [Oryzias latipes]  (COUP-TF-II-2) | 0 | 873.618 | 100.00% | 99.10% | 423 | 1 | 1 | 423 |
| cds.comp104218_c0_seq2\|m.161921 comp104218_c0_seq2\|g.161921 ORF comp104218_c0_seq2\|g.161921 comp104218_c0_seq2\|m.161921 type:complete len:418 (-) comp104218_c0_seq2:105-1358(-) | gi\|432861725\|ref\|XP_004069707.1\| | PREDICTED: COUP transcription factor 2-like isoform 1 [Oryzias latipes]  (COUP-TF-II-1) | 0 | 860.907 | 100.00% | 99.00% | 417 | 1 | 1 | 417 |
| cds.comp104805_c0_seq1\|m.178375 comp104805_c0_seq1\|g.178375 ORF comp104805_c0_seq1\|g.178375 comp104805_c0_seq1\|m.178375 type:complete len:601 (+) comp104805_c0_seq1:250-2052(+) | gi\|432932501\|ref\|XP_004081770.1\| | PREDICTED: nuclear receptor subfamily 4 group A member 2 [Oryzias latipes]  (NR4A2) | 0 | 1160.21 | 100.00% | 98.00% | 598 | 1 | 1 | 600 |
| cds.comp105150_c0_seq9\|m.186792 comp105150_c0_seq9\|g.186792 ORF comp105150_c0_seq9\|g.186792 comp105150_c0_seq9\|m.186792 type:complete len:611 (-) comp105150_c0_seq9:1760-3592(-) | gi\|432945337\|ref\|XP_004083548.1\| | PREDICTED: estrogen receptor-like [Oryzias latipes]  (ER) | 0 | 986.097 | 99.68% | 82.80% | 618 | 1 | 1 | 610 |
| cds.comp105176_c0_seq1\|m.187745 comp105176_c0_seq1\|g.187745 ORF comp105176_c0_seq1\|g.187745 comp105176_c0_seq1\|m.187745 type:5prime_partial len:335 (-) comp105176_c0_seq1:1573-2577(-) | gi\|432854604\|ref\|XP_004067983.1\| | PREDICTED: nuclear receptor subfamily 2 group F member 6-like [Oryzias latipes]  (NR2F6) | 0 | 640.573 | 82.27% | 96.70% | 406 | 73 | 1 | 334 |
| cds.comp105306_c2_seq5\|m.190579 comp105306_c2_seq5\|g.190579 ORF comp105306_c2_seq5\|g.190579 comp105306_c2_seq5\|m.190579 type:complete len:569 (-) comp105306_c2_seq5:875-2581(-) | gi\|432859606\|ref\|XP_004069177.1\| | PREDICTED: nuclear receptor subfamily 1 group D member 1-like [Oryzias latipes]  (NR1D1) | 0 | 932.169 | 100.00% | 85.20% | 568 | 1 | 1 | 568 |
| cds.comp105382_c0_seq3\|m.192214 comp105382_c0_seq3\|g.192214 ORF comp105382_c0_seq3\|g.192214 comp105382_c0_seq3\|m.192214 type:complete len:539 (-) comp105382_c0_seq3:92-1708(-) | gi\|432869184\|ref\|XP_004071664.1\| | PREDICTED: nuclear receptor subfamily 5 group A member 2 [Oryzias latipes]  (NR5A2) | 0 | 842.417 | 100.00% | 77.00% | 536 | 1 | 1 | 538 |
| cds.comp105702_c1_seq5\|m.202643 comp105702_c1_seq5\|g.202643 ORF comp105702_c1_seq5\|g.202643 comp105702_c1_seq5\|m.202643 type:5prime_partial len:594 (+) comp105702_c1_seq5:3-1784(+) | gi\|432888946\|ref\|XP_004075101.1\| | PREDICTED: retinoic acid receptor RXR-alpha-A [Oryzias latipes]  (RXRα-A) | 0 | 890.952 | 96.18% | 93.20% | 471 | 19 | 138 | 593 |
